# Influence of Rootstock Genotype and Ploidy Level on Common Clementine (*Citrus clementina* Hort. ex Tan) Tolerance to Nutrient Deficiency

**DOI:** 10.3389/fpls.2021.634237

**Published:** 2021-04-08

**Authors:** Julie Oustric, Stéphane Herbette, Raphaël Morillon, Jean Giannettini, Liliane Berti, Jérémie Santini

**Affiliations:** ^1^CNRS, Équipe de Biochimie et Biologie Moléculaire du Végétal, UMR 6134 SPE, Université de Corse, Corsica, France; ^2^UCA, INRAE, PIAF, Clermont-Ferrand, France; ^3^Equipe SEAPAG, CIRAD, UMR AGAP, Petit-Bourg, Guadeloupe, France; ^4^AGAP, University of Montpellier, CIRAD, INRAE, Institut Agro, Montpellier, France

**Keywords:** antioxidant, *Citrus*, nutrient deficiency, oxidative stress, photosynthesis, polyploidy, grafting

## Abstract

Nutrient deficiency, in particular when this involves a major macronutrient (N, P, and K), is a limiting factor on the performance of plants in their natural habitat and agricultural environment. In the citrus industry, one of the eco-friendliest techniques for improving tolerance to biotic and abiotic stress is based on the grafting of a rootstock and a scion of economic interest. Scion tolerance may be improved by a tetraploid rootstock. The purpose of this study was to highlight if tolerance of a common clementine scion (C) (*Citrus clementina* Hort. ex Tan) to nutrient deficiency could be improved by several diploid (2×) and their tetraploid (4×) counterparts citrus genotypes commonly used as rootstocks: Trifoliate orange × Cleopatra mandarin (C/PMC2x and C/PMC4x), Carrizo citrange (C/CC2x and C/CC4x), Citrumelo 4475 (C/CM2x and C/CM4x). The allotetraploid FlhorAG1 (C/FL4x) was also included in the experimental design. The impact of nutrient deficiency on these seven scion/rootstock combinations was evaluated at root and leaf levels by investigating anatomical parameters, photosynthetic properties and oxidative and antioxidant metabolism. Nutrient deficiency affects foliar tissues, physiological parameters and oxidative metabolism in leaves and roots in different ways depending on the rootstock genotype and ploidy level. The best known nutrient deficiency-tolerant common clementine scions were grafted with the doubled diploid Citrumelo 4475 (C/CM4x) and the allotetraploid FlhorAG1 (C/FL4x). These combinations were found to have less foliar damage, fewer changes of photosynthetic processes [leaf net photosynthetic rate (*P*_*net*_), stomatal conductance (*g*_*s*_), transpiration (E), maximum quantum efficiency of PSII (*F*_*v*_/*F*_*m*_), electron transport rate (ETR), ETR/*P*_*net*_], and effective quantum yield of PSII [*Y*(II)], less malondialdehyde accumulation in leaves and better functional enzymatic and non-enzymatic antioxidant systems. Common clementine scions grafted on other 4× rootstocks did not show better tolerance than those grafted on their 2× counterparts. Chromosome doubling of rootstocks did not systematically improve the tolerance of the common clementine scion to nutrient deficiency.

## Introduction

Citrus fruit crops represent an important economic activity worldwide. In recent years, easy peeler fruits including mandarins and its related varieties such as clementines have acquired a special place in global citrus trade at the expense of oranges, lemons, and pomelos. Citrus fruit crops require a significant amount of fertilizer to ensure satisfactory production and good quality fruit. In the current context of agro-ecological transition, a move must be made toward sustainable agriculture by reducing the use of farm inputs (fertilizers, crop protection products). A more moderate use of fertilizers would make it possible to respond to two constraints, (i) the economic constraint of the high costs of farm inputs and (ii) the ecological constraint due to the negative impact on water and soil biodiversity of excess fertilizer products not assimilated by plants.

Essential nutrients can be divided into two groups; macro- (N, K, P, Ca, Mg, and S) and micro-nutrients (Zn, Cu, Fe, Mn, B, Mo, Cl, and Ni). These play many roles such as: (i) essential metabolites (e.g., proteins, enzymes and coenzymes, cell walls, and chlorophyll), (ii) enzyme activators or regulators of enzyme-associated processes and (iii) non-structural factors in physiological processes (i.e., membrane integrity, photosynthesis, stomatal movement and environmental signaling) (Marschner, 1995; Grusak, 2001). The lack of one of these nutrients unbalances mineral element homeostasis which is essential for plant growth and optimum development. Firstly, plant nutrient deficiency limits most of the factors determining plant performance in terms of production per hectare and food quality (Tewari, 2004). When the deficiency becomes more pronounced, visible symptoms appear on the leaves, fruits and roots, indicating plant malfunction (Ericsson, 1995; Srivastava, 2013).

To cope with nutrient deficiencies, the plant must detect nutrient levels in the surrounding soil and within its cells and regulate various processes such as uptake, metabolism, remobilization and sequestration that lead to nutrient accumulation in the cell (Schachtman and Shin, 2007; Ohkama-Ohtsu and Wasaki, 2010; Aibara and Miwa, 2014).

As for other environmental stresses, plants under nutrient deficiency must cope with an unbalanced redox status that disturbs physiological and biochemical processes and leads to a disruption of growth and development (Law et al., 2001; Tewari et al., 2007; Yu-Chuan et al., 2008; Oustric et al., 2019; Zhang et al., 2019; Gong et al., 2020; Patel et al., 2020). Cells suffer oxidative damage due to the accumulation of reactive oxygen species (ROS), including superoxide (O_2_^•–^), singlet oxygen (^1^O_2_), radical hydroxyl (OH^•^) and hydrogen peroxide (H_2_O_2_). The highly reactive nature of these compounds causes severe damage to proteins and nucleic acids, as well as membrane leakage through lipid peroxidation (Mittler, 2002; Apel and Hirt, 2004; Mittler et al., 2004). Under environmental constraints, plants trigger the activity of antioxidant enzymes such as superoxide dismutase (SOD), catalase (CAT), ascorbate peroxidase (APX), or dehydroascorbate reductase (DHAR) and also the production of non-enzymatic compounds such as ascorbate and proline.

To improve tolerance to biotic and abiotic stresses, citrus cultivation relies on associations between a rootstock and a graft of economic interest. The roots store nitrogen and carbon reserves and absorb and assimilate nutrients from the soil before transferring them to the aerial parts via the xylem vessels. Nutrients and water uptake may increase in grafted plants due to the improved vigor of the rootstock root system which is one of the main reasons for the use of rootstocks in agriculture (Lee, 1994). These rootstock effects were also observed in citrus (Taylor and Dimsey, 1993; Lu et al., 2019).

Currently, most of the genetic resources of citrus rootstocks are diploid, i.e., they have two sets of chromosomes in their genetic heritage. Stress tolerance may be further improved by using tetraploid rootstocks. Indeed, much research has shown that the use of tetraploid rootstocks may be an effective way of improving stress tolerance in citrus trees, including water, salt and cold stress (Saleh et al., 2008; Allario et al., 2013; Oustric et al., 2017). Tetraploids (4×) resulting from incomplete mitosis of somatic embryos may occur naturally or artificially in seedlings with diploid (2×) apomictic genotypes (Cameron and Frost, 1968). Depending on the composition of their genomes and the mechanism of their formation, polyploids can be classified into two groups: autopolyploids that occur as a result of genome duplication or fusion of unreduced gametes (2*n*) within the same species, and allopolyploids formed by interspecific hybridization followed by chromosome doubling, fusion of unreduced gametes of two different species or spontaneous chromosomal doubling after hybridization, or by interspecific hybridization between two autotetraploid species.

Many *Citrus* genotypes used as rootstocks exist for clementine cultivation. Genotypes belong either to the *Citrus* volkameriana genus or are derived by hybridization between *Citrus* and *Poncirus* genus progenitors such as Citrumelo 4475, Carrizo citrange, and Trifoliate orange × Cleopatra mandarin. Volkameriana is widely used as it is adapted to dry, calcareous and saline soils and tolerant to tristeza, cachexia, and exocortis (Jacquemond et al., 2013). Citrumelo 4475 is used for its tolerance to tristeza and because it imparts cold tolerance to the graft (Jacquemond et al., 2013). Trifoliate orange × Cleopatra mandarin is adapted to calcareous, humid and saline soils and confers tolerance to several diseases including Tristeza and to cold conditions. Carrizo citrange is widely used in acidic and neutral soils and inherited Tristeza tolerance from its Trifoliate orange progenitor. Conversely, its poor performance in drought conditions limits its use in dry areas.

In a previous study, we compared the tolerance to nutritional deficiency of these 4 *citrus* seedling genotypes. In order to test the positive effect of polyploidization on the tolerance of seedlings to nutrient deficiency, their 4× counterparts were also studied (Oustric et al., 2019). The allotetraploid FlhorAG1, a somatic hybrid of Trifoliate orange and Willow leaf mandarin, was also included in the experimental design (Ollitrault et al., 2000). FlhorAG1 allotetraploid and Citrumelo 4475 doubled diploid appeared to be more tolerant than the other genotypes to prolonged nutrient deficiency, as shown by the lower reduction in photosynthesis parameters, and reduced accumulation of oxidation markers. This study was performed on seedlings and the value of these genotypes for the culture of citrus fruit requires studying their behavior as rootstocks.

The general working hypothesis that we wanted to check was that the use of a tetraploid rootstock could confer the scion a better tolerance under nutrient deficiency. Thus, the main objective of this paper was to highlight if several diploid and tetraploid citrus genotypes commonly used as rootstocks would improve the tolerance of a common clementine scion to nutrient deficiency. If so, technical itineraries requiring fewer inputs could be proposed taking into account the best scion/rootstock combination. The effect of nutrient deficiency was investigated on common clementine (C) (*Citrus clementina* Hort. ex Tan) grafted on 2× common rootstocks used in citrus cultivation and their 4× counterparts: the Trifoliate orange × Cleopatra mandarin hybrid (C/PMC2x and C/PMC4x), Citrumelo 4475 (C/CM2x and C/CM4x), Carrizo citrange (C/CC2x and C/CC4x). The allotetraploid FlhorAG1 (C/FL4x) was also included in the experimental design. The effects of nutrient deficiency on the clementine’s tolerance were evaluated at root and leaf levels by investigating anatomical parameters, photosynthetic properties and oxidative and antioxidant metabolism.

## Materials and Methods

### Plant Material and Growth Conditions

The experiment was carried out at the AREFLEC experimental station located in San Giuliano, Corsica (41° 47′ 27′′N and 09° 23′ 40′′E). Seedlings of 2× Trifoliate orange × Cleopatra mandarin (*Poncirus trifoliata* L. Raf. × *Citrus reshni* Hort. ex Tan.), Citrumelo 4475 (*Citrus paradisi* L. Macf. × *P. trifoliata* L. Raf.), Carrizo citrange (*Citrus sinensis* L. Osb. × *P. trifoliata* L. Raf.) and their three 4× counterparts were used as source materials. The FlhorAG1 (*Poncirus trifoliata* L. Raf. + *Citrus reticulata* Ten.), an allotetraploid form, was also included. For each of the seven genotypes, six plants were selected (giving a total of 42 plants) among seedlings made with seeds from trees maintained in the citrus germplasm collection (BCR NF 96-S-900 Citrus INRA/CIRAD) at San Giuliano, Corsica (France). The ploidy status of 2× and 4× seedlings was checked by 10-color flow cytometry (Partec I, Germany) as described by Froelicher et al. (2007). Clonal propagation by nucellar embryogenesis was verified by genotyping using SSR markers as described by Vieira et al. (2016). All 1-year seedlings were then grafted with a common clementine scion (*Citrus clementina* Hort. ex Tan; SRA 92) (Table 1) in 2013.

**TABLE 1:** Description of scion/rootstock combinations.

Scion	Rootstock	Varieties	Abbreviations	Ploidy level
	*Poncirus trifoliata* L. Raf. ×	Trifoliate orange ×	C/PMC	2×
	
	*Citrus reshni* Hort. ex Tan.	Cleopatra mandarin		4×
	
*Citrus clementina* Hort. ex Tan	*Poncirus trifoliata* L. Raf. + *Citrus reticulata* Ten.	FlhorAG1	C/FL	4×
	
	*Citrus paradisi* L. Macf. ×	Citrumelo 4475	C/CM	2×
	
	*Poncirus trifoliata* L. Raf.			4×
	
	*Citrus sinensis* L. Osb. ×	Carrizo citrange:	C/CC	2×
	
	*Poncirus trifoliata* L. Raf.	Oranger whashington navel × Trifoliate orange		4×

The 42 selected plants were potted in vermiculite and grown in a fertigation system in a tunnel greenhouse. Each scion/rootstock combination was then watered by two drippers (1 L/hr). The stock solution used for irrigation included 20-5-10 NPK + 2MgO fertilizer + trace components, in agreement with the recommendations of the local department of agriculture. The 42 plants were divided into two blocks treated by different levels of soil-less fertigation. A total of three plants of each variety was randomized by fertigation level. The two fertigation levels were the reference fertilization (control plants, 1 g/L) and irrigation water not supplemented with any nutrient inputs. The fertigation solutions were distributed by metering pumps. The vermiculite was washed for 48 h in order to eliminate all the nutritional reserves, prior to the start of the nutrient deficiency experiment. This avoided the latency phase that may have occurred if any fertilizer remaining in the vermiculite had been completely consumed by the plants.

According to a previous experiment of Oustric et al. (2019), samples and physiological measurements on leaves and roots were carried out from May 2018 to January 2019 at three different times (days): 0 (D0; control plant) and 210 (D210) days after the start of nutritional deprivation, and after 30 days of recovery (30DR). Leaf measurements were made and samples taken from homogeneous plants comprising four branches with leaves that had reached full maturity. For the root samples, the genotypes were removed from their pots, rinsed with water and then primary and secondary roots were collected at the same time for each scion/rootstock combination.

### Assessment of Leaf Damage

All the scion/rootstock combinations were ranked visually according to the degree of damage after 210 days of nutrient deficiency. Nutrient deficiency foliar tissue damage (chlorosis or shriveled leaves) was estimated on the five most representative and fully expanded leaves for each scion/rootstock combination (*n* = 15). Leaves were scored using a 0–3 rating scale, where 0 indicated the complete absence of symptoms, 1 light green leaves, 2 light green leaves with veins clearing and 3 shriveled yellow leaves.

### Foliar Mineral Analysis

Nutrient contents were measured on three sample for each scion/rootstock combination, i.e., one per tree, obtained by pooling eight fully-expanded leaves (*n* = 3) collected between 10:00 and 11:00 am. The leaves were cleaned with deionized water, dried at 65 ± 10°C in an oven overnight and transferred into a desiccator until cool. The dehydrated leaves were then sent to a CIRAD laboratory (Montpellier, France) for analysis of macro- and micro-nutrients.

Nutrient contents (phosphorus (P), potassium (K), calcium (Ca), magnesium (Mg), and sodium (Na), iron (Fe), zinc (Zn), boron (B), copper (Cu), and manganese (Mn) in the leaves were studied using an Agilent 720 simultaneous ICP-OES after double calcination including silica removal by adding hydrofluoric acid.

Lead total nitrogen (N) content was determined by combustion using a Leco TruMac N determinator.

### Gas Exchange Measurements

Leaf net photosynthetic rate (*P*_*net*_), stomatal conductance (*g*_*s*_) and transpiration rate (E) were measured using using a portable photosynthetic system (LCPro-SD, ADC Bioscientific, Hoddesdon, United Kingdom). The carbon dioxide concentration (CO_2_) in the leaf chamber was 380 μmol.mol^–1^, airflow rate was 500 μmol.s^–1^, light intensity was 1,400 μmol.m^–2^.s^–1^ and temperature 25°C. The measurements were made on three fully developed leaves for each scion/rootstock combination (*n* = 3) at between 7:00 and 11:00 am.

### Measurements of Chlorophyll *a* Fluorescence

Chlorophyll *a* fluorescence parameters were monitored with an OS1p (Opti-Sciences, Inc., Hudson, NH, United States). Measurements were made on three fully developed leaves for each scion/rootstock combination (*n* = 3) at between 7:00 and 11:00 am. Leaves were dark-adapted for 30 min using lightweight leaf clips to measure the minimal level of fluorescence (*F*_*o*_) followed by the maximal fluorescence (*F*_*m*_) after 1 s of a saturating flash (3,000 μmol photon⋅m^–2^⋅s^–1^) by an array of three light-emitting diodes (650 nm). Maximum fluorescence [*F*_*v*_/*F*_*m*_ = (*F*_*m*_ − *F*_*o*_)/*F*_*m*_] was calculated from these data according to Maxwell and Johnson (2000). Leaves were exposed to actinic light to evaluate the current fluorescence yield (*F*_*s*_) and the actual light-adapted fluorescence (*F*_*m*_′). The effective quantum yield of PSII [*Y*(II)], the electron transport rate (ETR) and the non-photochemical quenching coefficient [*Y*(NPQ)] were then calculated according to Baker (2008), using the following equations:

(1) $$Y(II)=(F_{m}^{\prime }-F_{s})/F_{m}^{\prime }$$

(2) $$Y(NPQ)=(F_{s}/F_{m}^{\prime })-Y(NO)$$

The ETR thought PSII [ETR(II)] was calculated according to Schreiber et al. (1995), using the equation:

(3) E⁢T⁢R⁢(I⁢I)=Y⁢(I⁢I)×P⁢A⁢R×0.5×0.84

The ETR/*P*_*net*_ ratio was calculated to estimate the use of electrons in other processes not related to the photosynthetic CO_2_ assimilation rate.

### Determination of Oxidative Stress and Antioxidant Levels

Biochemical analyses were performed on three samples for each scion/rootstock combination, i.e., one per tree, obtained by pooling eight fully-expanded leaves (*n* = 3) and three samples for each scion/rootstock combination, i.e., one per tree, obtained by pooling an equal weight of primary and secondary roots collected (*n* = 3) between 10:00 and 11:00 am. Samples were immediately immersed in liquid nitrogen and then stored at −80°C. Immediately prior to biochemical analysis, each leaf and root sample was ground to a fine powder in liquid nitrogen.

Assays of malondialdehyde (MDA), ascorbate and antioxidant enzymes (SOD, CAT, APX and dehydroascorbate reductase) were performed as described by Santini et al. (2013).

Hydrogen peroxide (H_2_O_2_) was assayed using the PeroxiDetect kit (Sigma-Aldrich), which is based on the oxidation of ferrous (Fe^2+^) to ferric (Fe^3+^) ions at acidic pH. The subsequent reaction of Fe^3+^ ions forms a blue adduct with xylenol orange (3,3′-bis[*N*,*N*-bis(carboxymethyl)aminomethyl] *o*-cresolsulfonephthalein, sodium salt) visible at 560 nm. Proline content was measured as described by Oustric et al. (2019).

A V-630 spectrophotometer was used for all measurements (Jasco Inc., Tokyo, Japan).

### Statistical Analyses

All statistical measurements were performed with R statistical software (v.2.12.1)^1^ and the Rcmdr package. The qualitative factors studied were sampling date (days) (D0 and D210 after nutrient deficiency, and 30DR of recovery for leaves and roots), the common clementine scion grafted onto rootstocks and subjected to nutrient deficiency (C/PMC, C/FL, C/CC, and C/CM, for leaves and roots) and the ploidy level of nutrient stressed rootstocks (C/PMC2x and C/PMC4x, C/FL4x, C/CM2x and C/CM4x, C/CC2x and C/CC4x for leaves and roots). The impact of these three factors was analyzed using a two-way ANOVA followed by LSD test at *p* < 0.05.

The data for gas exchange, chlorophyll fluorescence, antioxidant parameters and oxidative markers obtained at D210 of nutrient deficiency and after 30DR of recovery for clementine scions grafted onto the seven rootstocks were analyzed by hierarchical group analysis and heatmaps generated by Heatmap.2 function of the gplot package 3.0.1 for Rstudio (v.1.3.1093)^2^.

## Results

In order to minimize any effects due to changes in environmental conditions (photoperiod, temperature in the tunnel greenhouse, etc.) when comparing the responses to nutrient deficiency of different genotypes, results on stressed scion/rootstock combinations were expressed as ratios relative to the values obtained for controls. Thus, only the effect of the nutrient deficiency was taken into account.

### Leaf Damage

**FIGURE 1:**
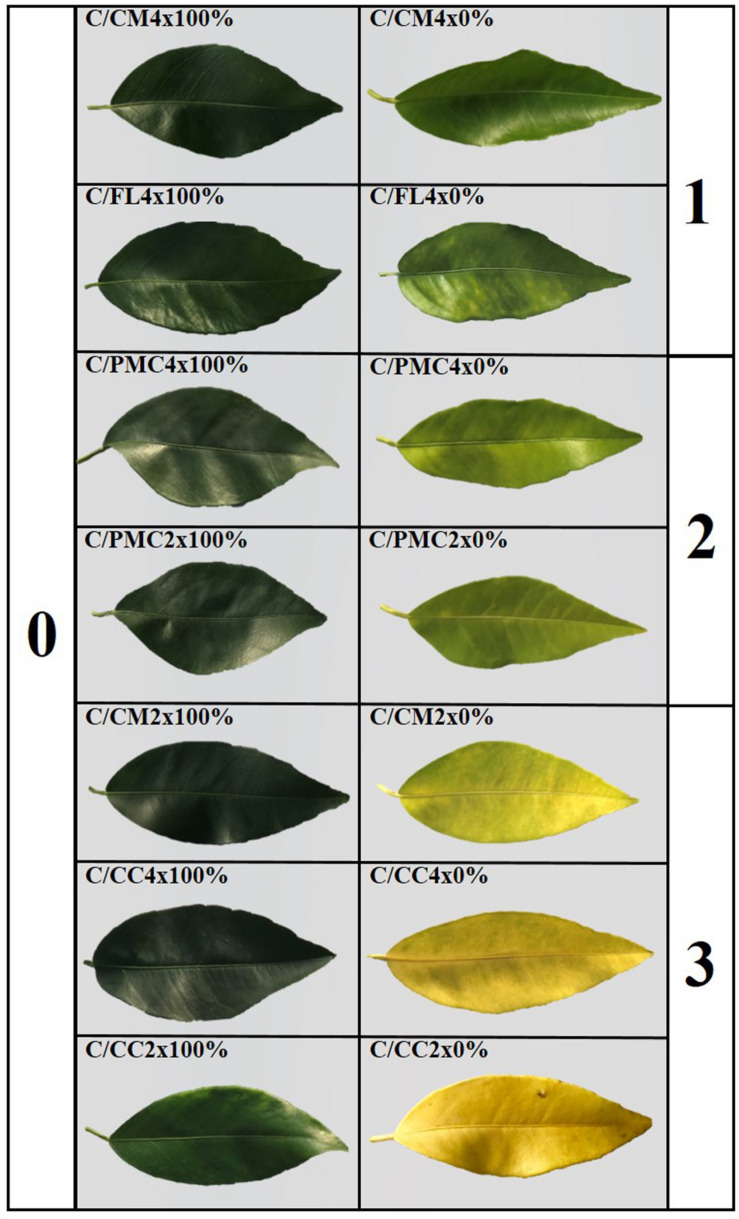
Assessment of leaf damages after 210 days of total nutrient deficiency (0%) on clementine trees grafted onto the seven rootstocks compared to controls (100%). Genotypes are ranked based on the leaf symptoms from the lesser affected (1) to the more affected (3).

The most representative level of leaf damage induced after 210 days of nutrient stress in each rootstock/scion combination is shown in Figure 1. Level 0 on our rating scale corresponded to the control with green leaves and no disease symptoms. Minimum foliar tissue damage (level l) was observed in C/CM4x and C/FL4x with light green leaves. C/PMC4x and C/PMC2x had light green leaves with yellow veins (level 2). Mean foliar tissue damage was higher in C/CM2x, C/CC2x and C/CC4x with yellow leaves (level 3).

### Change in Macro- and Micro- Nutrients

Overall after 210 days of nutrient stress, there was a decrease in foliar N, Fe (except C/CC4x), Cu (except C/PMC4x), Mn, and B of all scion/rootstock combinations (Table 2 and Supplementary Tables 1, 2). An increase or similar values was found for the P, K (except C/CC2x), Mg, Ca (except C/CC4x), Na (except C/PMC2x) and Zn (except C/CM4x, C/CC4x and C/CC2x) (Table 2 and Supplementary Tables 1, 2).

**TABLE 2:** Total leaf contents of macronutrient in common clementine scion grafted onto the seven rootstocks.

Parameters	Days	C/PMC4x	C/PMC2x	C/FL4x	C/CM4x	C/CM2x	C/CC4x	C/CC2x
N (%)	*D0*	*3.03 ± 0.25*^*a*^	*2.94 ± 0.19*^a^	*3.00 ± 0.16*^*a*^	*3.91 ± 0.57*^*a*^	*3.32 ± 0.04*^*a*^	*3.40 ± 0.44*^*a*^	*2.99 ± 0.15*^*a*^
……………………………………………………………………………………………………………………………………………………………..								
N (ratio)	D210	0.40 ± 0.02^a^	0.37 ± 0.03^ab^	0.35 ± 0.02^ab^	0.43 ± 0.09^a^	0.40 ± 0.02^a^	0.40 ± 0.01^a^	0.33 ± 0.02^b^

	30DR	0.37 ± 0.01^b^	0.38 ± 0.05^ab^	0.35 ± 0.01^b^	0.31 ± 0.08^c^	0.34 ± 0.06^b^	0.45 ± 0.01^a^	0.40 ± 0.02^ab^
P (%)	*D0*	*0.16 ± 0.09*^*ab*^	*0.14 ± 0.01*^*ab*^	*0.15 ± 0.01*^*ab*^	*0.15 ± 0.01*^*ab*^	*0.16 ± 0.01*^*ab*^	*0.19 ± 0.01*^*a*^	*0.15 ± 0.02*^*ab*^
……………………………………………………………………………………………………………………………………………………………..								
P (ratio)	D210	1.97 ± 0.15^a^	1.27 ± 0.17^b^	2.03 ± 0.15^a^	1.30 ± 0.19^b^	2.13 ± 0.15^a^	1.53 ± 0.12^b^	1.37 ± 0.07^b^

	30DR	2.90 ± 0.13^a^	1.71 ± 0.13^b^	1.05 ± 0.26^c^	1.59 ± 0.05^b^	1.70 ± 0.08^b^	2.18 ± 0.15^ab^	2.03 ± 0.01^ab^
K (%)	*D0*	*2.51 ± 0.02*^*a*^	*2.15 ± 0.14*^*ab*^	*2.53 ± 0.28*^*a*^	*2.34 ± 0.22*^*ab*^	*1.79 ± 0.08*^*b*^	*2.21 ± 0.01*^*ab*^	*2.60 ± 0.15*^*a*^
……………………………………………………………………………………………………………………………………………………………..								
K (ratio)	D210	1.16 ± 0.04^b^	1.43 ± 0.04^ab^	1.87 ± 0.02^a^	1.56 ± 0.09^ab^	1.46 ± 0.07^ab^	1.14 ± 0.05^b^	0.78 ± 0.15^c^

	30DR	1.87 ± 0.05^a^	1.01 ± 0.10^c^	1.56 ± 0.02^ab^	1.30 ± 0.01^b^	1.41 ± 0.07^ab^	1.55 ± 0.12^ab^	1.58 ± 0.12^ab^
Ca (%)	*D0*	*1.97 ± 0.05*^*a*^	*2.20 ± 0.06*^*a*^	*1.30 ± 0.13*^*b*^	*1.60 ± 0.22*^*ab*^	*1.67 ± 0.31*^*ab*^	*1.32 ± 0.15*^*b*^	*1.62 ± 0.12*^*ab*^
……………………………………………………………………………………………………………………………………………………………..								
Ca (ratio)	D210	1.19 ± 0.10^ab^	1.03 ± 0.13^b^	1.08 ± 0.06^b^	0.91 ± 0.05^b^	1.39 ± 0.01^a^	0.84 ± 0.06^bc^	0.96 ± 0.07^b^

	30DR	1.15 ± 0.14^b^	1.09 ± 0.08^b^	0.98 ± 0.16^b^	1.04 ± 0.02^b^	1.50 ± 0.03^a^	1.11 ± 0.06^b^	1.16 ± 0.09^b^
Mg (%)	*D0*	*0.68 ± 0.02*^*b*^	*0.62 ± 0.05*^*b*^	*0.66 ± 0.07*^*b*^	*0.88 ± 0.02*^*a*^	*0.54 ± 0.04*^*b*^	*0.69 ± 0.07*^*b*^	*0.62 ± 0.02*^*b*^
……………………………………………………………………………………………………………………………………………………………..								
Mg (ratio)	D210	1.15 ± 0.08^b^	1.19 ± 0.03^b^	1.17 ± 0.01^b^	1.69 ± 0.05^a^	1.63 ± 0.16^a^	1.33 ± 0.09^ab^	1.10 ± 0.11^b^

	30DR	1.44 ± 0.07^a^	1.48 ± 0.03^a^	1.27 ± 0.07^ab^	0.80 ± 0.03^b^	1.47 ± 0.09^a^	1.22 ± 0.09^ab^	1.21 ± 0.16^ab^
Na (%)	*D0*	*0.014 ± 0.001*^*a*^	*0.019 ± 0.002*^*a*^	*0.017 ± 0.001*^*a*^	*0.018 ± 0.002*^*a*^	*0.014 ± 0.002*^*a*^	*0.014 ± 0.001*^*a*^	*0.016 ± 0.002*^*a*^
……………………………………………………………………………………………………………………………………………………………..								
Na (ratio)	D210	0.980 ± 0.125^d^	0.842 ± 0.100^d^	4.464 ± 0.036^a^	1.500 ± 0.178^bc^	1.166 ± 0.066^c^	4.119 ± 0.094^a^	2.000 ± 0.121^b^
	30DR	2.500 ± 0.179^ab^	1.333 ± 0.209^b^	1.307 ± 0.154^b^	1.111 ± 0.101^b^	1.409 ± 0.079^b^	3.750 ± 0.120^a^	2.526 ± 0.058^ab^

*Data shown at D210 and 30DR are means (n = 3 ± standard error) expressed as ratios with respect to the values obtained on control which have not been subjected to stress. For further informations, means values of control data (D0) (n = 3 ± standard error) were also included and indicated by italics characters. Data were analysed using ANOVA and Fisher LSD tests (P < 0.05). Different letters indicate significant differences between genotypes along the time course. D0, control, D210, 210 days after the start of nutritional deprivation, and 30DR, after 30 days of recovery.*

After 30 days of recovery, N, Fe (except in C/CC4x and C/CM2x), Cu (except in C/CC2x), Mn (except in C/CM4x) and B contents were lower and P contents were higher than the control values in all scion/rootstock combinations. K, Ca, Mg (except C/CM4x) and Na contents were higher or similar than control values for all scion/rootstock combinations (Table 2 and Supplementary Tables 1, 2). Zn contents were similar in C/CC4x, C/CM2x and C/CM4x or lower in C/CC2x, C/PMC2x, C/PMC4x and C/FL4x than control values (Supplementary Table 2).

### Physiological and Biochemical Responses of Scion/Rootstock Combinations to Nutrient Deficiency and Recovery

**FIGURE 2:**
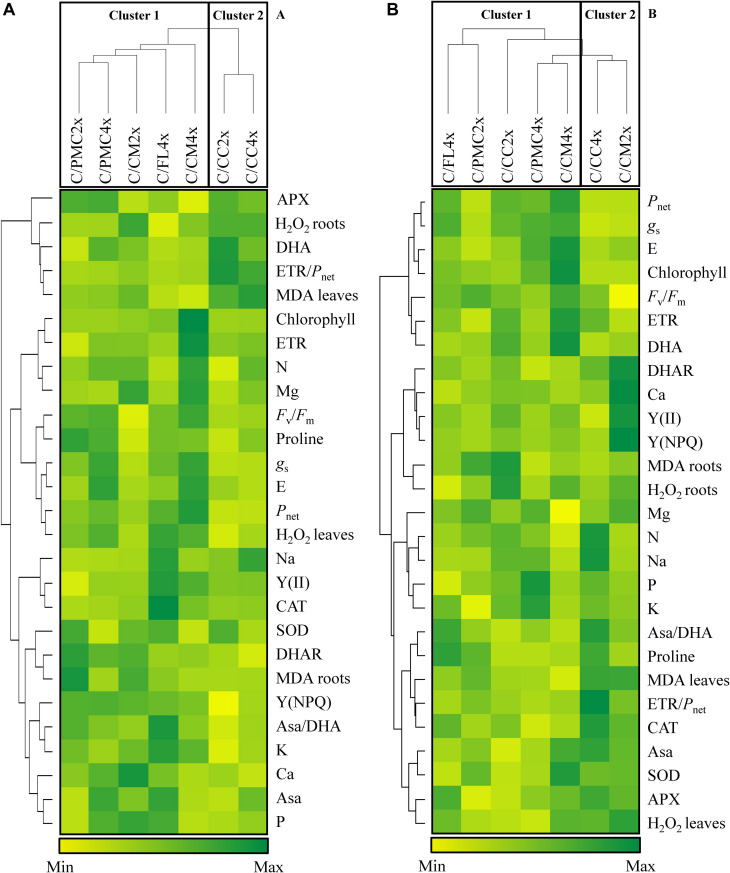
Hierarchical cluster analysis and heatmap displaying mineral contents and parameters responding to nutrient deficiency in different scion/rootstock combinations after 210 days of nutrient deficiency (D210) **(A)** and after 30 days of recovery (30DR) **(B)**. Values are means of 3 independent (*n* = 3) measurements for each parameter. Hierarchical cluster analysis dispatches the seven scion/rootstock combinations based on the different parameters. The heatmap shows the differences between the seven scion/rootstock combinations and treatments for each parameter. Color scale shows the intensity of the normalized mean values of different parameters. Values are associated with color ranging from yellow (low) to dark green (high).

Based on photosynthesis and antioxidant parameters and oxidative markers visualized within the heatmap (Figures 2A,B), the seven scion/rootstock combinations could be separated into two groups.

After 210 days of nutrient deficiency, the first group comprised C/PMC4x, C/PMC2x, C/FL4x, C/CM4x and C/CM2x and the second group C/CC4x and C/CC2x. On the whole, the scion/rootstock combinations in group 1 were distinguished by a lower MDA and DHA content in leaves and H_2_O_2_ content in roots and a higher photosynthetic capacity [*P*_net_, *g*_s_, E, *F*_v_/*F*_m_, Y(NPQ)] (except for C/CM2x) and lower ETR/*P*_net_ than group 2.

After 30 days of recovery, C/CM2x and C/CC2x changed groups, respectively in groups 2 and 1. The first group comprised C/CC2x, C/PMC2x, C/FL4x, C/PMC4x and C/CM4x and the second group comprised C/CM2x and C/CC4x. The first group could be divided into 3 subgroups consisting of C/CC2x associated with C/PMC2x, C/PMC4x with C/FL4x, and C/CM4x alone. C/CM4x were characterized by a higher photosynthetic capacity (*P*_net_, *g*_s_, E, chlorophyll, *F*_v_/*F*_m_ratio, ETR) and lower foliar MDA content than C/CM2x and C/CC4x, whereas C/CC2x, C/PMC2x, C/FL4x and C/PMC4x had intermediate values for these variables. C/CM4x was distinguished by a higher Asa and SOD activity and higher DHA and H_2_O_2_ content in leaves and lower CAT and APX activities than the other scion/rootstock combinations.

### Change in Photosynthetic Capacities

At D210, a smaller decline in *P*_net_, *g*_s_ and E was observed in C/CM4x than in C/FL4x, C/PMC2x and C/PMC4x and, to a greater extent, in C/CM2x, C/CC2x and C/CC4x (Tables 3, 4 and Supplementary Tables 3, 4). C/CM4x showed a better recovery of *P*_net_ at 30DR than C/PMC4x, C/FL4x and C/CC2x and, to a greater extent, C/CM2x, C/CC4x and C/PMC2x. The decrease in *g*_s_ and E content was lower in C/CM4x and C/PMC4x than in C/PMC2x, C/FL4x, C/CM2x, C/CC2x and C/CC4x at D210. C/CM4x showed a low decrease in *g*_s_ and E content at 30DR (Table 3 and Supplementary Table 3). Concerning the decline in chlorophyll content, this was lower in C/CM4x and C/FL4x and C/PMC4x than in the other scion/rootstock combinations at D210 and 30DR, respectively (Table 3 and Supplementary Table 3).

**TABLE 3:** Leaf photosynthetic parameters of the seven scion/rootstock combinations after 210 days of nutrient deficiency (D210) and 30 days of recovery (30DR).

Parameters	Day	C/PMC4x	C/PMC2x	C/FL4x	C/CM4x	C/CM2x	C/CC4x	C/CC2x
*P*_net_ (μmol CO_2_.m^–2^.s^–1^)	*D0*	*7.807 ± 1.077*^*b*^	*9.247 ± 1.536*^*ab*^	*9.343 ± 0.948*^*ab*^	*10.368 ± 0.404*^*a*^	*10.352 ± 0.225*^*a*^	*9.482 ± 0.589*^*a*^	*10.106 ± 0.445*^*a*^
……………………………………………………………………………………………………………………………………………………………..								
*P*_net_(ratio)	D210	0.275 ± 0.004^*b*^	0.197 ± 0.044^bc^	0.319 ± 0.005^ab^	0.447 ± 0.020^a^	0.154 ± 0.019^c^	0.065 ± 0.002^d^	0.052 ± 0.004^d^

	30DR	0.222 ± 0.001^b^	0.051 ± 0.006^c^	0.248 ± 0.021^b^	0.350 ± 0.010^a^	0.061 ± 0.043^c^	0.056 ± 0.004^c^	0.249 ± 0.081^b^
*g*_s_ (mol CO_2_.m^–2^ .s^–1^)	*D0*	*0.133 ± 0.033*^*ab*^	*0.132 ± 0053*^*ab*^	*0.165 ± 0.031*^*a*^	*0.068 ± 0.019*^*cd*^	*0.057 ± 0.005*^*cd*^	*0.100 ± 0.019*^*bc*^	*0.018 ± 0.015*^*d*^
……………………………………………………………………………………………………………………………………………………………..								
*g*_s_(ratio)	D210	0.380 ± 0.034^a^	0.214 ± 0.074^b^	0.268 ± 0.029^b^	0.387 ± 0.002^a^	0.095 ± 0.008^c^	0.098 ± 0.007^c^	0.084 ± 0.008^c^

	30DR	0.255 ± 0.005^a^	0.092 ± 0.001^b^	0.260 ± 0.056^a^	0.278 ± 0.055^a^	0.077 ± 0.002^c^	0.060 ± 0.004^c^	0.220 ± 0.074^ab^
E (mmol H_2_O.m^–2^ .s^–1^)	*D0*	*0.867 ± 0.084*^*ab*^	*0.920 ± 0.007*^*ab*^	*0.788 ± 0.176*^*b*^	*0.904 ± 0.089*^*ab*^	*0.344 ± 0.027*^*c*^	*1.350 ± 0.148*^*a*^	*1.140 ± 0.211*^*a*^
……………………………………………………………………………………………………………………………………………………………..								
E (ratio)	D210	0.472 ± 0.214^a^	0.165 ± 0.017^c^	0.221 ± 0.024^b^	0.480 ± 0.005^a^	0.145 ± 0.025^cd^	0.112 ± 0.012^d^	0.184 ± 0.024^c^

	30DR	0.302 ± 0.012^b^	0.079 ± 0.001^d^	0.180 ± 0.023^c^	0.406 ± 0.007^a^	0.168 ± 0.025^c^	0.119 ± 0.004^cd^	0.156 ± 0.017^c^
Chlorophyll (DUALEX units)	*D0*	*36.15 ± 2.855*^*a*^	*40.883 ± 1.346*^*a*^	*45.95 ± 9.758*^*a*^	*45.133 ± 2.984*^*a*^	*43.867 ± 6.323*^*a*^	*45.217 ± 5.648*^*a*^	*42.95 ± 5.687*^*a*^
……………………………………………………………………………………………………………………………………………………………..								
Chlorophyll (ratio)	D210	0.131 ± 0.066^c^	0.130 ± 0.028^c^	0.218 ± 0.023^b^	0.628 ± 0.037^a^	0.169 ± 0.096^c^	0.142 ± 0.022^c^	0.129 ± 0.058^c^
	30DR	0.184 ± 0.009^b^	0.143 ± 0.009^c^	0.164 ± 0.019^bc^	0.254 ± 0.043^a^	0.114 ± 0.045^d^	0.113 ± 0.017^d^	0.133 ± 0.031^cd^

*Data shown at D210 and 30DR are means (n = 3 ± standard error) expressed as ratios with respect to the values obtained on control which have not been subjected to stress. For further informations, means values of control data (D0) (n = 3 ± standard error) were also included and indicated by italics characters. Data were analysed using ANOVA and Fisher LSD tests (P < 0.05). Different letters indicate significant differences between genotypes along the time course. D0, control, D210, 210 days after the start of nutritional deprivation, and 30DR, after 30 days of recovery.*

**TABLE 4:** Chlorophyll *a* fluorescence parameters of the seven scion/rootstock combinations after 210 days of nutrient deficiency (D210) and 30 days of recovery (30DR).

Parameters	Day	C/PMC4x	C/PMC2x	C/FL4x	C/CM4x	C/CM2x	C/CC4x	C/CC2x
*F*_v_/F_m_	*D0*	*0.825 ± 0.005*^*a*^	*0.821 ± 0.007*^*a*^	*0.82 ± 0008*^*a*^	*0.825 ± 0.008*^*a*^	*0.826 ± 0.003*^*a*^	*0.771 ± 0.083*^*a*^	*0.785 ± 0.044*^*a*^
……………………………………………………………………………………………………………………………………………………………..								
*F*_v_/F_m_ (ratio)	D210	0.751 ± 0.060^ab^	0.712 ± 0.040^ab^	0.633 ± 0.023^b^	0.820 ± 0.046^a^	0.269 ± 0.018^d^	0.492 ± 0.088^c^	0.450 ± 0.029^c^

	30DR	0.491 ± 0.040^bc^	0.665 ± 0.012^ab^	0.581 ± 0.021^b^	0.712 ± 0.068^a^	0.242 ± 0.065^c^	0.552 ± 0.016^b^	0.572 ± 0.011^b^
ETR (μmol .e^–1^.m^–2^ .s^–1^)	*D0*	*31.05 ± 2.936*^*a*^	*28.667 ± 4.256*^*a*^	*30.983 ± 6.126*^*a*^	*31.05 ± 5.365*^*a*^	*29.183 ± 8.800*^*a*^	*25.367 ± 3.006*^*a*^	*26.817 ± 6.963*^*a*^
……………………………………………………………………………………………………………………………………………………………..								
ETR (ratio)	D210	0.573 ± 0.025^bc^	0.308 ± 0.118^d^	0.470 ± 0.037^c^	0.972 ± 0.022^a^	0.567 ± 0.026^bc^	0.587 ± 0.023^bc^	0.537 ± 0.088^bc^

	30DR	0.444 ± 0.012^c^	0.263 ± 0.043^d^	0.585 ± 0.012^bc^	1.054 ± 0.005^a^	0.325 ± 0.046^d^	0.732 ± 0.011^b^	0.792 ± 0.101^b^
ETR/*P*_net_	*D0*	*3.977 ± 0.442*^*a*^	*3.100 ± 0.903*^*ab*^	*3.316 ± 0.322*^*ab*^	*2.995 ± 0.327*^*ab*^	*2.819 ± 0.923*^*ab*^	*2.675 ± 0.139*^*b*^	*2.654 ± 0.721*^*b*^
……………………………………………………………………………………………………………………………………………………………..								
ETR/*P*_net_(ratio)	D210	2.123 ± 0.173^bc^	1.563 ± 0.053^c^	1.473 ± 0.018^c^	2.176 ± 0.112^bc^	3.683 ± 0.002^b^	9.026 ± 0.777^a^	11.805 ± 0.593^a^

	30DR	2.005 ± 0.237^d^	5.157 ± 0.569^b^	2.376 ± 0.020^d^	3.011 ± 0.197^c^	5.322 ± 0.118^b^	13.066 ± 0.198^a^	3.181 ± 0.464^c^
Y(II)	*D0*	*0.624 ± 0.084*^*a*^	*0.570 ± 0.041*^*a*^	*0.616 ± 0.085*^*a*^	*0.617 ± 0.106*^*a*^	*0.580 ± 0.174*^*a*^	*0.470 ± 0.113*^*a*^	*0.558 ± 0.087*^*a*^
……………………………………………………………………………………………………………………………………………………………..								
Y(II) (ratio)	D210	0.574 ± 0.025^c^	0.225 ± 0.103^d^	1.192 ± 0.109^a^	0.952 ± 0.034^ab^	0.567 ± 0.026^c^	0.707 ± 0.065^bc^	0.674 ± 0.136^bc^

	30DR	0.432 ± 0.031^bc^	0.406 ± 0.054^bc^	0.519 ± 0.140^b^	0.587 ± 0.038^b^	0.977 ± 0.029^a^	0.250 ± 0.008^c^	0.667 ± 0.025^ab^
Y(NPQ)	*D0*	*0.186 ± 0.009*^*a*^	*0.047 ± 0.003*^*b*^	*0.055 ± 0.014*^*b*^	*0.046 ± 0.005*^*b*^	*0.052 ± 0.005*^*b*^	*0.048 ± 0.010*^*b*^	*0.047 ± 0.010*^*b*^
……………………………………………………………………………………………………………………………………………………………..								
Y(NPQ) (ratio)	D210	1.721 ± 0.221^a^	1.684 ± 0.201^a^	1.510 ± 0.272^a^	1.383 ± 0.171^a^	1.623 ± 0.035^a^	1.074 ± 0.230^a^	0.469 ± 0.062^b^
	30DR	1.279 ± 0.038^c^	0.819 ± 0.045^d^	1.731 ± 0.251^bc^	2.539 ± 0.504^b^	10.329 ± 0.359^a^	0.715 ± 0.151^d^	2.846 ± 0.019^b^

*Data shown at D210 and 30DR are means (n = 3 ± standard error) expressed as ratios with respect to the values obtained on control which have not been subjected to stress. For further informations, means values of control data (D0) (n = 3 ± standard error) were also included and indicated by italics characters. Data were analysed using ANOVA and Fisher LSD tests (P < 0.05). Different letters indicate significant differences between genotypes along the time course. D0, control, D210, 210 days after the start of nutritional deprivation, and 30DR, after 30 days of recovery.*

A smaller decrease in *F*_v_/*F*_m_ was observed in C/CM4x, C/PMC2x, C/PMC4x and C/FL4x than in the other scion/rootstock combinations at D210 and in C/CM4x and C/PMC2x at 30DR (Table 4 and Supplementary Table 4). Conversely to the other scion/rootstock combinations, ETR in C/CM4x remained below the control at D210 and 30DR (Table 4 and Supplementary Table 4). Overall, ETR/*P*_net_ increased in all scion/rootstock combinations but this increase was greater in C/CM2x, C/CC2x and C/CC4x and in C/CC4x, C/PMC2x and C/CM2x than in the other scion/rootstock combinations at D210 and 30DR, respectively (Table 4 and Supplementary Table 4). Although Y(II) remained stable in C/FL4x and C/CM4x, it decreased in the other scion/rootstock combinations and, in particular, in C/PMC2x at D210 (Table 4 and Supplementary Table 4). However, Y(II) decreased in all scion/rootstock combinations, except in C/FL4x and C/CM4x and C/CM2x, where it remained stable at D210 and 30DR, respectively (Table 4 and Supplementary Table 4). Y(NPQ) increased or stayed close to 1 in all scion/rootstock combinations except in C/CC2x at D210 and C/CC4x and C/PMC2x at 30DR (Table 4 and Supplementary Table 4).

### Change in Antioxidant Molecules

The Asa contents and Asa/DHA ratio increased or remained stable in all scion/rootstock combinations at D210 (Table 5 and Supplementary Table 5). C/FL4x presented a larger increase Asa/DHA ratio than the other scion/rootstock combinations at D210. Asa content and Asa/DHA ratio decreased at 30DR in all scion/rootstock combinations or only in C/CC2x and C/CM4x, respectively.

**TABLE 5:** Antioxidant activities of the seven scion/rootstock combinations after 210 days of nutrient deficiency (D210) and 30 days of recovery (30DR).

Parameters	Day	C/PMC4x	C/PMC2x	C/FL4x	C/CM4x	C/CM2x	C/CC4x	C/CC2x
Asa (μmol .g^–1^ FW)	*D0*	*1.461 ± 0.096*^*ab*^	*1.437 ± 0.106*^*ab*^	*1.530 ± 0.037*^*ab*^	*0.853 ± 0.322*^*b*^	*1.398 ± 0.025*^*ab*^	*1.674 ± 0.178*^*a*^	*1.561 ± 0.087*^*ab*^
……………………………………………………………………………………………………………………………………………………………..								
Asa (ratio)	D210	3.852 ± 0.386^a^	1.885 ± 0.229^c^	3.978 ± 0.183^a^	2.087 ± 0.086^c^	2.833 ± 0.172^b^	3.090 ± 0.142^b^	1.881 ± 0.182^c^

	30DR	0.507 ± 0.030^a^	0.574 ± 0.059^a^	0.503 ± 0.069^a^	0.699 ± 0.147^a^	0.639 ± 0.143^a^	0.737 ± 0.056^a^	0.416 ± 0.069^a^
Asa/DHA	*D0*	*2.068 ± 0.157*^*a*^	*2.810 ± 1.309*^*a*^	*2.437 ± 0.067*^*a*^	*1.356 ± 0.066*^*a*^	*2.390 ± 0.303*^*a*^	*2.189 ± 0.294*^*a*^	*2.188 ± 0.219*^*a*^
……………………………………………………………………………………………………………………………………………………………..								
Asa/DHA (ratio)	D210	3.494 ± 0.428^c^	4.756 ± 0.168^b^	6.439 ± 0.052^a^	3.158 ± 0.126^c^	2.910 ± 0.302^c^	2.536 ± 0.305^cd^	1.086 ± 0.086^d^

	30DR	1.511 ± 0.425^ab^	1.416 ± 0.082^ab^	3.364 ± 0.228^a^	0.654 ± 0.053^b^	1.832 ± 0.024^ab^	3.862 ± 0.429^a^	0.508 ± 0.009^b^
Proline (μmol .g^–1^ FW)	*D0*	*12.986 ± 0.413*^*c*^	*12.026 ± 0.570*^*c*^	*15.217 ± 0.353*^*a*^	*14.677 ± 0.231*^*ab*^	*13.685 ± 0.275*^*bc*^	*15.421 ± 0.576*^*a*^	*13.487 ± 0.446*^*bc*^
……………………………………………………………………………………………………………………………………………………………..								
Proline (ratio)	D210	0.460 ± 0.078^a^	0.506 ± 0.015^a^	0.395 ± 0.010^ab^	0.382 ± 0.010^ab^	0.242 ± 0.006^c^	0.363 ± 0.014^b^	0.256 ± 0.003^c^

	30DR	0.321 ± 0.129^c^	0.479 ± 0.081^ab^	0.563 ± 0.130^a^	0.345 ± 0.009^bc^	0.359 ± 0.023^bc^	0.536 ± 0.097^a^	0.319 ± 0.115^c^
SOD (U .mg^–1^ protein)	*D0*	*3.734 ± 0.107*^*c*^	*6.238 ± 0.186*^*a*^	*3.773 ± 0.127*^*c*^	*2.766 ± 0.023*^*d*^	*2.996 ± 0.062*^*d*^	*5.113 ± 0.211*^*b*^	*3.097 ± 0.007*^*cd*^
……………………………………………………………………………………………………………………………………………………………..								
SOD (ratio)	D210	0.591 ± 0.019^c^	0.750 ± 0.062^a^	0.734 ± 0.021^a^	0.594 ± 0.077^c^	0.707 ± 0.038^a^	0.624 ± 0.036^b^	0.734 ± 0.021^a^

	30DR	0.350 ± 0.016^c^	0.476 ± 0.027^b^	0.308 ± 0.019^c^	0.597 ± 0.017^a^	0.472 ± 0.001^b^	0.458 ± 0.018^b^	0.309 ± 0.019^c^
CAT (μmol.min^–1^ protein)	*D0*	*1.309 ± 0.015*^*ab*^	*1.052 ± 0.015*^*b*^	*1.318 ± 0.213*^*ab*^	*1.414 ± 0.004*^*a*^	*1.010 ± 0.075*^*b*^	*0.382 ± 0.003*^*c*^	*0.941 ± 0.008*^*b*^
……………………………………………………………………………………………………………………………………………………………..								
CAT (ratio)	D210	0.479 ± 0.024^c^	0.281 ± 0.003^d^	3.538 ± 0.544^a^	1.299 ± 0.109^b^	0.818 ± 0.090^bc^	0.890 ± 0.030^bc^	0.778 ± 0.051^bc^

	30DR	0.331 ± 0.004^d^	1.157 ± 0.021^c^	2.371 ± 0.325^b^	0.951 ± 0.111^c^	2.398 ± 0.330^b^	3.566 ± 0.291^a^	1.836 ± 0.112^bc^
APX (μmol.min^–1^ protein)	*D0*	*1.742 ± 0.066*^*a*^	*1.564 ± 0.083*^*ab*^	*1.310 ± 0.009*^*cd*^	*1.487 ± 0.003*^*bc*^	*1.633 ± 0.006*^*ab*^	*1.187 ± 0.009*^*d*^	*1.161 ± 0.006*^*d*^
……………………………………………………………………………………………………………………………………………………………..								
APX (ratio)	D210	1.985 ± 0.053^a^	1.934 ± 0.177^a^	1.564 ± 0.050^b^	1.118 ± 0.002^c^	1.330 ± 0.063^ab^	1.728 ± 0.016^a^	1.898 ± 0.015^a^

	30DR	2.442 ± 0.269^a^	2.048 ± 0.112^a^	2.778 ± 0.383^a^	2.615 ± 0.316^a^	2.664 ± 0.014^a^	2.84 ± 0.233^a^	2.173 ± 0.254^a^
DHAR (μmol.min^–1^ protein)	*D0*	*43.744 ± 0.118*^*b*^	*46.157 ± 0.323*^*a*^	*22.757 ± 0.056*^*c*^	*22.127 ± 0.677*^*c*^	*19.18 ± 0.133*^*d*^	*19.188 ± 0.366*^*d*^	*21.455 ± 1.248*^*cd*^
……………………………………………………………………………………………………………………………………………………………..								
DHAR (ratio)	D210	0.581 ± 0.003^b^	0.693 ± 0.025^a^	0.452 ± 0.005^c^	0.469 ± 0.014^c^	0.610 ± 0.009^b^	0.322 ± 0.019^d^	0.420 ± 0.017^c^
	30DR	0.371 ± 0.004^b^	0.415 ± 0.019^b^	0.453 ± 0.005^ab^	0.410 ± 0.063^b^	0.602 ± 0.010^a^	0.495 ± 0.013^ab^	0.476 ± 0.022^ab^

*Data shown at D210 and 30DR are means (n = 3 ± standard error) expressed as ratios with respect to the values obtained on control which have not been subjected to stress. For further informations, means values of control data (D0) (n = 3 ± standard error) were also included and indicated by italics characters. Data were analysed using ANOVA and Fisher LSD tests (P < 0.05). Different letters indicate significant differences between genotypes along the time course. D0, control, D210, 210 days after the start of nutritional deprivation, and 30DR, after 30 days of recovery.*

The proline content was lower than the control in all scion/rootstock combinations at D210 and 30DR, but this reduction was lower in C/CC2x at D210 and C/PMC4x and C/CC2x at 30DR (Table 5 and Supplementary Table 5).

### Change in Enzymatic Antioxidants

SOD and DHAR decreased in all scion/rootstock combinations at D210 and 30DR (Table 5 and Supplementary Table 5). The decrease in SOD was greater in C/CM4x and C/PMC4x at D210 and in C/PMC4x, C/FL4x and C/CC2x at 30DR than in the other scion/rootstock combinations.

DHAR decreased more severely in C/CC4x, C/CC2x, C/CM4x and C/FL4x at D210 and in C/CM4x, C/PMC4x and C/PMC2x at 30DR in comparison to the other scion/rootstock combinations. CAT increased in C/FL4x and in lower extent in C/CM4x, whereas it decreased in the other scion/rootstock combinations at D210 (Table 5 and Supplementary Table 5). Only C/PMC4x stayed below the control value at 30DR. APX was the only enzymatic activity to remain greater than 1 in all scion/rootstock combinations at D210 and 30DR (Table 5 and Supplementary Table 5). APX activity was greater in C/PMC2x, C/PMC4x, C/CC2x and C/CC4x than in the other scion/rootstock combinations at D210, whereas there were no significant differences between the combinations at 30DR.

### Change in Oxidative Markers in Leaves and Roots

At D210, the MDA content increased in leaves of all scion/rootstock combinations. On the contrary, no accumulation was observed for DHA content in leaves (except in C/PMC4x and C/CC2x) and MDA in roots (except C/PMC2x) and H_2_O_2_ in leaves (Table 6 and Supplementary Table 6). H_2_O_2_ increased in roots of C/CM2x, C/CC4x and C/CC2x but decreased in the other scion/rootstock combinations.

**TABLE 6:** Oxidative markers of the seven scion/rootstock combinations after 210 days of nutrient deficiency (D210) and 30 days of recovery (30DR).

Parameters	Day	C/PMC4x	C/PMC2x	C/FL4x	C/CM4x	C/CM2x	C/CC4x	C/CC2x
DHA (μmol .g^–1^ FW)	*D0*	*0.709 ± 0.067*^*a*^	*0.571 ± 0.193*^*a*^	*0.675 ± 0.012*^*a*^	*0.631 ± 0.056*^*a*^	*0.592 ± 0.082*^*a*^	*0.792 ± 0.178*^*a*^	*0.720 ± 0.100*^*a*^
……………………………………………………………………………………………………………………………………………………………..								
DHA	D210	1.248 ± 0.428^ab^	0.387 ± 0.168^d^	0.564 ± 0.052^cd^	0.665 ± 0.126^cd^	0.978 ± 0.302^bc^	1.071 ± 0.305^bc^	1.697 ± 0.086^a^
	30DR	0.346 ± 0.070^b^	0.337 ± 0.082^b^	0.257 ± 0.228^bc^	1.148 ± 0.053^a^	0.335 ± 0.024^b^	0.193 ± 0.088^c^	0.795 ± 0.009^ab^
MDA leaves (nmol .g^–1^ FW)	*D0*	*7.582 ± 1.324*^*c*^	*3.093 ± 0.700*^*c*^	*39.943 ± 5.297*^*a*^	*20.478 ± 4.536*^*b*^	*23.077 ± 2.643*^*b*^	*21.622 ± 4.526*^*b*^	*24.494 ± 5.424*^*b*^
……………………………………………………………………………………………………………………………………………………………..								
MDA leaves	D210	2.190 ± 0.033^bc^	2.080 ± 0.286^bc^	1.550 ± 0.202^c^	1.242 ± 0.085^d^	2.645 ± 0.146^b^	3.530 ± 0.159^a^	2.994 ± 0.080^ab^
	30DR	0.885 ± 0.097^c^	1.704 ± 0.085^ab^	1.144 ± 002^bc^	0.289 ± 0.052^d^	2.180 ± 0.143^a^	2.230 ± 0.250^a^	0.922 ± 0.051^bc^
H_2_O_2_ leaves (nmol .g^–1^ FW)	*D0*	*60.096 ± 3.086*^*c*^	*64.293 ± 3.535*^*bc*^	*62.605 ± 3.634*^*bc*^	*79.600 ± 5.645*^*a*^	*73.948 ± 3.085*^*ab*^	*66.795 ± 3.667*^*b*^	*79.524 ± 3.858*^*a*^
……………………………………………………………………………………………………………………………………………………………..								
H_2_O_2_ leaves	D210	1.008 ± 0.007^a^	0.900 ± 0.050^b^	1.080 ± 0.046^a^	1.007 ± 0.175^a^	0.777 ± 0.059^bc^	0.789 ± 0.036^bc^	0.671 ± 0.045^c^
	30DR	0.563 ± 0.046^d^	0.684 ± 0.103^c^	0.991 ± 0.174^b^	1.080 ± 0.07^b^	1.278 ± 0.047^a^	1.055 ± 0.039^b^	0.631 ± 0.006^c^
MDA roots (nmol .g^–1^ FW)	*D0*	2.818 ± 0.036^*c*^	*3.256 ± 0.067*^*bc*^	*5.724 ± 0.019*^*b*^	*10.328 ± 0.846*^*a*^	*5.946 ± 0.027*^*b*^	*5.185 ± 0.110*^*b*^	*5.272 ± 0.080*^*b*^
……………………………………………………………………………………………………………………………………………………………..								
MDA roots	D210	0.661 ± 0.034^c^	1.313 ± 0.043^a^	0.770 ± 0.001^b^	0.620 ± 0.106^c^	1.089 ± 0.070^a^	0.633 ± 0.027^c^	0.615 ± 0.051^c^
	30DR	0.430 ± 0.171^e^	1.452 ± 0.099^ab^	0.784 ± 0.065^bc^	0.748 ± 0.039^c^	0.854 ± 0.086^b^	0.589 ± 0.021^d^	1.716 ± 0.128^a^
H_2_O_2_ roots (nmol .g^–1^ FW)	*D0*	*22.271 ± 1.900*^*b*^	*22.201 ± 4.466*^*b*^	*31.459 ± 1.748*^*a*^	*23.793 ± 1.347*^*b*^	*23.425 ± 0.516*^*b*^	*21.224 ± 0.949*^*b*^	*11.536 ± 1.257*^*c*^
……………………………………………………………………………………………………………………………………………………………..								
H_2_O_2_ roots	D210	0.713 ± 0.021^b^	0.703 ± 0.043^b^	0.400 ± 0.089^c^	0.866 ± 0.046^b^	1.244 ± 0.017^a^	1.134 ± 0.063^a^	1.130 ± 0.046^a^
	30DR	0.638 ± 0.178^c^	0.801 ± 0.051^b^	0.295 ± 0.046^d^	1.227 ± 0.030^ab^	1.200 ± 0.050^ab^	0.752 ± 0.078^b^	1.627 ± 0.058^a^

*Data shown at D210 and 30DR are means (n = 3 ± standard error) expressed as ratios with respect to the values obtained on control which have not been subjected to stress. For further informations, means values of control data (D0) (n = 3 ± standard error) were also included and indicated by italics characters. Data were analysed using ANOVA and Fisher LSD tests (P < 0.05). Different letters indicate significant differences between genotypes along the time course. D0, control, D210, 210 days after the start of nutritional deprivation, and 30DR, after 30 days of recovery.*

At 30DR, only C/CM4x showed an increase in DHA content (Table 6 and Supplementary Table 6). MDA accumulation in leaves was lower in C/CM4x than in C/PMC4x, C/CC2x and C/FL4x and much lower than in C/PMC2x, C/CM2x and C/CC4x. The H_2_O_2_ content in leaves increased in C/CM2x or stayed stable in C/FL4x, C/CM4x and C/CC4x whereas it decreased in the other scion/rootstock combinations at 30DR (Table 6 and Supplementary Table 6). Unlike the other scion/rootstock combinations, C/PMC2x and C/CC2x showed an increase in MDA content in roots at 30DR (Table 6 and Supplementary Table 6). H_2_O_2_ content increased in roots of C/CC2x, C/CM2x and C/CM4x and but decreased in the other scion/rootstock combinations (Table 6 and Supplementary Table 6).

## Discussion

### Comparison of the Sensitivity to Nutrient Deficiency of Clementine Scions Grafted on Different Rootstocks Genotypes Varying in Their Ploidy Level

Total nutrient deficiency induced chlorosis as shown by visual changes in color from yellow to light green in the whole plant of each scion/rootstock combination (Figure 1). Chlorosis is a characteristic symptom of nutritional deficiency, especially N deficiency, which is accompanied by the degradation of chlorophyll and the internal chloroplast structure altering the photosynthetic process (Lambers et al., 2008; Boussadia et al., 2010). Chlorosis was associated with a similar large decrease in N after 210 days of nutrient deficiency in all scion scion/rootstock combinations (Figure 1 and Table 2) (Elavarasan and Premalatha, 2019). The increases in the P, K, and Mg contents in leaves of scion/rootstock combinations could be explained by a concentration effect due to the loss of nitrogen or their absorption from other anatomical parts of the plant (roots or leaves) (Tewari et al., 2007). This similar effect of nutrient deficiency on mineral contents cannot explain the differences in yellowing between scion/rootstock combinations. Yellowing variations might be related to a variation in chlorophyll oxidation as a consequence of an excess of ROS generated by nutrient deficiency (Praveen and Gupta, 2018; Qi et al., 2019). Depending on their genotype and level of ploidy, the rootstocks have different impacts on the regulation of oxidative stress rather than the regulation of minerals in the scion. To our knowledge, there are no data on the tolerance to total nutrient deficiency of the scion/rootstock combinations studied in this experiment.

In this study, scion/rootstock combinations were ranked according to their sensitivity to nutrient deprivation according to leaf damage and MDA content at D210 of nutrient deprivation (Figure 1 and Table 6). Leaf symptom is a phenotypic response to nutrient deficiency used to rank plants (Srivastava, 2013; Oustric et al., 2019). C/CM4x and C/FL4x were ranked as “tolerant” and C/PMC2x and C/PMC4x as “moderately tolerant” because their leaves had level 1 and level 2 damage, respectively, with less MDA accumulation than the other scion/rootstock combinations. On the contrary, the level 3 leaf symptom and high MDA accumulation in C/CM2x, C/CC4x, and C/CC2x indicted their “sensitivity” to total nutrient deficiency.

### What Are the Reasons for This Difference in Tolerance to Nutrient Deficiency Between Scion/Rootstocks Combinations?

The nutritional deficiency implemented for 210 days had a negative effect on all rootstock/scion combinations.

The decrease in N content would have affected photosynthetic capacity and chlorophyll *a* fluorescence (Terashima and Evans, 1988; Nunes et al., 1993; Amtmann and Armengaud, 2009; Jin et al., 2015). In fact, half of foliar N is allocated to the photosynthetic apparatus (Makino and Osmond, 1991). “Moderately tolerant” C/PMC2x and C/PMC4x and “tolerant” C/FL4x and C/CM4x (group 1) showed fewer changes of the photosynthetic process than “sensitive” scion/rootstock combinations, as evidenced by the lower decrease in *P*_*net*_, *g*_*s*_, E, *F*_*v*_/*F*_*m*_, ETR, ETR/*P*_*net*_, and *Y*(II) (Figure 2A and Tables 3, 4). *P*_*net*_ can be affected by stomatal and/or non-stomatal factors (Weng and Hsu, 2001). As indicated by the synergy and lower reduction in *P*_*net*_, *g*_*s*_, and E, “moderately tolerant” C/PMC2x and C/PMC4x and “tolerant” C/FL4x and C/CM4x (Figure 2A and Table 3) implement a more effective tolerance strategy than “sensitive” scion/rootstock, despite N decrease. This would be explained by a closure of stomata to limit transpiration and thus water loss at foliar level (Green and Mitchell, 1992; Fracheboud et al., 1999; Afrousheh et al., 2010; Feng et al., 2012; Marguerit et al., 2012). In addition, the higher *P*_*net*_ in “tolerant” C/CM4x and C/FL4x could be due to a lesser impact of N decrease in their chlorophyll content than in the other scion/rootstock associations (Fleischer, 1935). Their differences in *P*_*net*_ could be linked to a decrease in Rubisco concentrations and/or Calvin cycle enzyme also known to be affected during N deprivation (Ferrar and Osmond, 1986; Evans and Terashima, 1987).

A decrease in chlorophyll content associated with a disturbance in chlorophyll fluorescence parameters [*F*_*v*_/*F*_*m*_, ETR, ETR/*P*_*net*_, and *Y*(II)] suggests that N decrease negatively influenced the efficiency of e^–^ capture (Evans and Terashima, 1987; Grassi et al., 2001; Kumar and Goh, 2002; Tikkanen et al., 2014; Cetner et al., 2017). The impact on the “tolerant” C/CM4x genotype was lower than for the other scion/rootstock combinations. The ETR/*P*_*net*_ ratio is indicative of increased electron consumption diverted to photorespiration or to alternative processes (Flexas et al., 1999, 2002). During electron transport, ROS are formed by the consecutive one-electron reduction of O_2_ and by the concerted two-electron oxidation of H_2_O on the PSII electron acceptor and donor sides, respectively (Pospíšil, 2009). In the heatmap at D210, ETR/*P*_*net*_ may have been associated with the increase in MDA (Figure 2A and Tables 4, 6) which is a marker for oxidation. These results indicate that the formation of ROS is due to the excess of electrons for the carbon reduction process (Pan et al., 2006). Moreover, the decrease in photosynthetic capacity in all scion/rootstock combinations after D210 of nutrient deficiency is due to damage to the whole photosystem II complex and particularly to chloroplast structure following the action of oxidative molecules (Papadakis et al., 2004; Guerfel et al., 2009; Paparnakis et al., 2013). The synergy between *Y*(NPQ), Asa content and Asa/DHA ratio, with the increase Asa content and Asa/DHA ratio, may optimize protection against photoinhibition by enhancing non-photochemical quenching [*Y*(NPQ)] in all scion/rootstock combinations, except in the “sensitive” C/CC2x (Figure 2A and Tables 4, 5) (Talla et al., 2011; Karpinska et al., 2018).

After 30 days of recovery, C/CM4x (group 1) had higher values for photosynthesis parameters (*P*_*net*_, *g*_*s*_, E, *F*_*v*_/*F*_*m*_, and ETR) and a lower accumulation of MDA and H_2_O_2_ in leaves (Figure 2B and Tables 3, 4, 6). C/CM4x seemed to recover more rapidly with a better reversibility of the damage caused by nutrient deficiency compared to the other scion/rootstock combinations.

On the whole, rootstock autotetraploidization does not systematically improve the scion’s tolerance to nutrient deficiency. C/CM4x is the only combination with a 4× rootstock that showed a significant improvement in tolerance compared to its homologous 2× rootstock after 210 days of nutrient deficiency and 30 days of recovery. These results are in agreement with those obtained by Oustric et al. (2019) who showed that ungrafted CM4x seedlings were more tolerant than CM2x seedlings. Conversely, C/PMC4x and C/PMC2x adapted better to nutrient deficiency than their non-grafted counterparts. The compatibility of the PMC rootstock with common clementine scion can be explained by their close genetic heritage (Jacquemond et al., 2013), as they both have the mandarin (*Citrus reticulata*) as parent. When considering allotetraploidisation, non-grafted FL4x showed a higher resistance than other non-grafted genotypes like CM4x in our previous study (Oustric et al., 2019). In the present study, C/FL4x appeared to present the same level of tolerance as C/CM4x when grafted.

### Does the Antioxidant System Help Improve Tolerance to Nutrient Deficiency in Both Roots and Leaves?

The antioxidant capacity of the scion and rootstock during nutrient deficiency differed with rootstock genotype. Whatever the sensitivity of the rootstock/scion combination, DHAR and SOD activities did not seem to play a prominent role during nutrient deficiency (Table 5). These results did not agree with the large increases in SOD and DHAR activities observed in the same ungrafted rootstocks (Oustric et al., 2019). Although DHAR activity decreased, it appeared to be sufficient (except in C/PMC4x and C/CC2x) to maintain a lower or similar DHA content to the control while increasing the Asa/DHA ratio and Asa content after D210 (Table 5). Conversely, different changes in APX and CAT activity (Table 5) were observed. While CAT activity was only greater than controls in C/CM4x and C/FL4x, APX activity was greater than controls in all scion/rootstock combinations. The high APX activity is consistent with the large increase in Asa required for its function in all scion/rootstock combinations. The complementary metabolic role of CAT and APX suggested by their highly contrasted *K*_*m*_ for H_2_O_2_ (Mhamdi et al., 2012) would explain the low H_2_O_2_ and MDA values in “tolerant” C/CM4x and C/FL4x. Although proline decreased in all scion/rootstock combinations (Tables 5, 6), it might be sufficient to complement the CAT activity in maintaining low H_2_O_2_ and MDA values in “tolerant” C/FL4x and C/CM4x (Kishor and Dange, 1990; Ozden et al., 2009). In the other scion/rootstock combinations, whatever their tolerance level, H_2_O_2_ contents were close to or below those of the control (Table 6) and were accompanied by a larger increase in MDA than in C/CM4x and C/FL4x. Unlike MDA, H_2_O_2_ is a transient molecule that accumulates over time to reveal different levels of oxidative damage between scion/rootstock combinations. These results suggest that APX activity is active on H_2_O_2_ but insufficient to prevent the formation of OH^•^ and therefore of compounds induced by lipid peroxidation such as MDA.

The high CAT and APX activity in “tolerant” C/CM4x and C/FL4x associated with a high ascorbate content indicates the importance of functional collaboration between enzymatic and non-enzymatic molecules for an effective antioxidant system (Blokhina et al., 2003). These results were consistent with their lower decrease in photosynthetic capacity (Tables 3, 4).

At root level, MDA and H_2_O_2_ levels were similar or lower than the control values for all scion/rootstock combinations (Table 6) indicating an increase in the tolerance of the root system to nutrient deficiency regardless of the rootstock genotype and ploidy level. The root system appears to be less affected by mineral deficiency than the scion. Thus, differences in tolerance of scion/rootstock combinations appear to be due to the distinct influence of the rootstock depending on its genotype and ploidy level and on the performance of the scion.

During recovery, the activity of the antioxidant machinery was found to be similar in all scion/rootstock combinations. As during nutrient deficiency, SOD and DHAR activities were lower than controls but CAT and APX were similar or higher than controls depending on the scion/rootstock combination (Table 5). C/CC2x, C/PMC2x, C/PMC4x, C/FL4x and to a greater extent C/CM4x had lower MDA and H_2_O_2_ accumulation in leaves than C/CM2x and C/CC4x. This could be explained by their state of stress after D210 of nutrient deficiency (Table 6). C/CC2x seems to have a more effective antioxidant system than the “sensitive” C/CC4x and C/CM2x and this allows it to recover to a similar extent to the “tolerant” and “moderately tolerant” genotypes (Figure 2B and Table 5).

## Conclusion

The experimental design of our study revealed differences in tolerance to nutrient deficiency in clementine scions depending on rootstock genotype and ploidy level.

Among the most tolerant scion/rootstock combinations, the doubled diploid Citrumelo 4475 and the allotetraploid FlhorAG1, both grafted with a common clementine scion, were characterized by less foliar damage, fewer alterations in photosynthetic processes, a reduction in oxidative markers and better functional enzymatic and non-enzymatic antioxidant systems. The other common clementine scion grafted on 4× rootstocks did not show better tolerance than their 2× counterparts. The fact that Citrumelo 4475 4× and FlhorAG1 4× rootstocks reduce the damage caused by nutrient deficiency in common clementine scion suggests that they could be used in citrus orchards.

A lower and more rationale use of fertilizers would have a positive impact on the economy and on soil and water ecology while conserving growth and development. In the future, it would be interesting to test the impact of this 4× rootstock on growth, fruit production and clementine quality during nutrient deficiency. New emerging varieties such as triploids could also be tested. Recent citrus breeding programs have in fact mainly focused on these as they produce seedless fruit with a different maturity period and useful pomological, agronomical and organoleptic traits.

## Data Availability Statement

The raw data supporting the conclusions of this article will be made available by the authors, without undue reservation.

## Author Contributions

JO collected data, did the statistical analysis, interpreted the results, and drafted the manuscript. JS designed the study and drafted the manuscript. RM, SH, JG, and LB designed the study and helped to draft the manuscript. All authors contributed to the article and approved the submitted version.

## Conflict of Interest

The authors declare that the research was conducted in the absence of any commercial or financial relationships that could be construed as a potential conflict of interest.

## Footnotes

1 http://www.R-project.org

2 https://rstudio.com

## Supplementary Material

Supplementary Table 1 Means of macronutrient contents in leaves of the seven scion/rootstock combinations.

Supplementary Table 2 Means of micronutrient contents in leaves of the seven scion/rootstock combinations.

Supplementary Table 3 Means of gas exchange parameters and chlorophyll content of the seven scion/rootstock combinations.

Supplementary Table 4 Means of chlorophyll *a* fluorescence parameters of the seven scion/rootstock combinations.

Supplementary Table 5 Means of antioxidant parameters of the seven scion/rootstock combinations.

Supplementary Table 6 Means of oxidative markers of the seven scion/rootstock combinations.

## Abbreviations

2 ×: diploid
4 ×: doubled diploid
APX: ascorbate peroxidase
Asa: reduced ascorbate
Asa/DHA: redox status of ascorbate
C/CC2x: diploid Carrizo citrange
C/CC4x: doubled diploid Carrizo citrange
CAT: catalase
C/CM2x: diploid Citrumelo 4475
C/CM4x: doubled diploid Citrumelo 4475
C/FL4x: allotetraploid FlhorAG1
C/PMC2x: diploid Trifoliate orange × Cleopatra mandarin
C/PMC4x: doubled diploid Trifoliate orange × Cleopatra mandarin
DHA: oxidized ascorbate
ETR: electron transport rate
*F*_*o*_: minimal chlorophyll a fluorescence
*F*_*m*_: maximal fluorescence
*F*_*m*_ ′: actual light-adapted fluorescence
*F*_*s*_: current fluorescence yield
*F*_*v*_: variable fluorescence
*F*_*v*_/*F*_*m*_: maximum quantum efficiency of PSII
*g*_*s*_: stomatal conductance
MDA: malondialdehyde
*P*_*net*_: leaf net photosynthetic rate
ROS: reactive oxygen species
SOD: superoxide dismutase
*Y*(NPQ): non-photochemical quenching coefficient
*Y*(II): effective quantum yield of PSII.
