# Supplementary material for: Influence of Rootstock Genotype and Ploidy Level on Common Clementine (Citrus clementina Hort. ex Tan) Tolerance to Nutrient Deficiency
Source: Front Plant Sci. 2021 Apr 8;12:634237. doi: 10.3389/fpls.2021.634237 (PMC8060649; doi:10.3389/fpls.2021.634237)
Supplement: Supplementary Table 1 — Means of macronutrient contents in leaves of the seven scion/rootstock combinations. [file Table_1.docx]

**Supplementary Table 1.** Means of macronutrient contents in leaves of the seven scion/rootstock combinations.

| Scion/rootstock combinations | Days | C/PMC4x | C/PMC2x | C/FL4x | C/CM4x | C/CM2x | C/CC4x | C/CC2x |
| --- | --- | --- | --- | --- | --- | --- | --- | --- |
| N (%) | D0-100% | 3.03 | 2.94 | 3 | 3.91 | 3.32 | 3.4 | 2.99 |
|  | D210-100% | 2.89 | 2.97 | 3.42 | 3.02 | 2.86 | 3.02 | 3.4 |
|  | D210-0% | 1.16 | 1.11 | 1.2 | 1.29 | 1.15 | 1.19 | 1.12 |
|  | 30DR-100% | 3.17 | 3.02 | 3.93 | 3.92 | 4.2 | 2.54 | 3.04 |
|  | 30DR-0% | 1.19 | 1.16 | 1.36 | 1.21 | 1.42 | 1.15 | 1.2 |
| P (%) | D0-100% | 0.16 | 0.14 | 0.15 | 0.15 | 0.16 | 0.19 | 0.15 |
|  | D210-100% | 0.14 | 0.14 | 0.14 | 0.16 | 0.08 | 0.25 | 0.31 |
|  | D210-0% | 0.27 | 0.18 | 0.29 | 0.21 | 0.17 | 0.38 | 0.43 |
|  | 30DR-100% | 0.08 | 0.16 | 0.31 | 0.18 | 0.12 | 0.15 | 0.16 |
|  | 30DR-0% | 0.24 | 0.28 | 0.33 | 0.29 | 0.21 | 0.32 | 0.32 |
| K (%) | D0-100% | 2.51 | 2.15 | 2.53 | 2.34 | 1.79 | 2.21 | 2.6 |
|  | D210-100% | 2.19 | 2.02 | 1.56 | 1.5 | 1.91 | 3.64 | 6.2 |
|  | D210-0% | 2.54 | 2.89 | 2.93 | 2.33 | 2.79 | 4.14 | 4.86 |
|  | 30DR-100% | 1.53 | 3.36 | 2.51 | 2.34 | 1.97 | 2.01 | 2.4 |
|  | 30DR-0% | 2.85 | 3.4 | 3.92 | 3.04 | 2.79 | 3.12 | 3.79 |
| Ca (%) | D0-100% | 1.95 | 2.2 | 1.3 | 1.65 | 1.67 | 1.32 | 1.62 |
|  | D210-100% | 1.78 | 2.17 | 1.43 | 1.74 | 1.18 | 1.69 | 1.93 |
|  | D210-0% | 2.12 | 2.24 | 1.54 | 1.59 | 1.64 | 1.42 | 1.86 |
|  | 30DR-100% | 1.43 | 2.34 | 1.47 | 1.65 | 1.01 | 1.79 | 1.95 |
|  | 30DR-0% | 1.64 | 2.54 | 1.44 | 1.71 | 1.52 | 1.99 | 2.26 |
| Mg (%) | D0-100% | 0.63 | 0.62 | 0.66 | 0.88 | 0.54 | 0.69 | 0.62 |
|  | D210-100% | 0.81 | 0.7 | 0.65 | 0.57 | 0.42 | 0.61 | 0.81 |
|  | D210-0% | 0.93 | 0.83 | 0.76 | 0.96 | 0.69 | 0.81 | 0.89 |
|  | 30DR-100% | 0.6 | 0.51 | 0.64 | 0.87 | 0.62 | 0.83 | 0.84 |
|  | 30DR-0% | 0.87 | 0.75 | 0.81 | 0.7 | 0.91 | 1.01 | 1.01 |
| Na (%) | D0-100% | 0.014 | 0.019 | 0.017 | 0.018 | 0.014 | 0.014 | 0.016 |
|  | D210-100% | 0.019 | 0.019 | 0.013 | 0.012 | 0.016 | 0.015 | 0.02 |
|  | D210-0% | 0.019 | 0.016 | 0.017 | 0.018 | 0.019 | 0.063 | 0.04 |
|  | 30DR-100% | 0.007 | 0.014 | 0.013 | 0.018 | 0.015 | 0.016 | 0.019 |
|  | 30DR-0% | 0.017 | 0.018 | 0.017 | 0.02 | 0.021 | 0.06 | 0.048 |

Values are means (*n* = 3 ± standard error) of three independent measurements from three samples for each genotype, i.e. one per tree, obtained by pooling 8 fully-expanded leaves. Data were analysed using ANOVA and Fisher LSD tests (P < 0.05). Scion/rootstock combinations grown in nutrient reference solution (100%) and without nutrient solution (0%) at the beginning of the experiment (D0); 210 days after the start of nutritional deprivation (D210), and after 30 days of recovery (30DR).
