# Supplementary material for: Influence of Rootstock Genotype and Ploidy Level on Common Clementine (Citrus clementina Hort. ex Tan) Tolerance to Nutrient Deficiency
Source: Front Plant Sci. 2021 Apr 8;12:634237. doi: 10.3389/fpls.2021.634237 (PMC8060649; doi:10.3389/fpls.2021.634237)
Supplement: Supplementary Table 2 — Means of micronutrient contents in leaves of the seven scion/rootstock combinations. [file Table_2.docx]

**Supplementary Table 2.** Means of micronutrient contents in leaves of the seven scion/rootstock combinations.

| Scion/rootstock combinations | Days | C/PMC4x | C/PMC2x | C/FL4x | C/CM4x | C/CM2x | C/CC4x | C/CC2x |
| --- | --- | --- | --- | --- | --- | --- | --- | --- |
| Fe (mg/kg) | D0-100% | 124.25 | 84.15 | 93.2 | 93.25 | 99.75 | 75 | 272.15 |
|  | D210-100% | 128.1 | 81.7 | 99.2 | 88.8 | 117.1 | 81.3 | 271.9 |
|  | D210-0% | 40.67 | 63.2 | 49.5 | 49.67 | 82.35 | 105.15 | 121.55 |
|  | 30DR-100% | 118.4 | 86.6 | 88.4 | 86.6 | 82.4 | 68.7 | 273.4 |
|  | 30DR-0% | 35.3 | 57.9 | 49.97 | 77.87 | 130.1 | 137.4 | 166.67 |
| Cu (mg/kg) | D0-100% | 13.03 | 13.75 | 16.6 | 14.57 | 14.9 | 13.17 | 10.6 |
|  | D210-100% | 13.85 | 13 | 16.05 | 15.9 | 13.35 | 13.8 | 10.25 |
|  | D210-0% | 13.3 | 10.8 | 11.87 | 10.77 | 11.57 | 10.4 | 7.55 |
|  | 30DR-100% | 12.75 | 14.5 | 16.2 | 17.7 | 14.75 | 12.65 | 10.7 |
|  | 30DR-0% | 9.5 | 7.23 | 12.4 | 10.75 | 12.23 | 11.07 | 15.35 |
| Mn (mg/kg) | D0-100% | 31.2 | 28.85 | 18.87 | 18.67 | 32.73 | 27.3 | 34.1 |
|  | D210-100% | 31.09 | 29.4 | 19.15 | 19.65 | 35.45 | 29.15 | 36.65 |
|  | D210-0% | 19.63 | 24.4 | 13.03 | 4.03 | 13.37 | 7.15 | 16.53 |
|  | 30DR-100% | 31.85 | 28.3 | 19.9 | 18.4 | 29.35 | 27.2 | 34.4 |
|  | 30DR-0% | 14.2 | 19.15 | 5.53 | 17.27 | 7.53 | 6.67 | 9.47 |
| B (mg/kg) | D0-100% | 108.2 | 77.4 | 123.65 | 108.8 | 75.9 | 60.95 | 87 |
|  | D210-100% | 110.67 | 68.8 | 130.67 | 104.2 | 65.4 | 58.2 | 90.3 |
|  | D210-0% | 35.63 | 25.6 | 48.23 | 22.8 | 22.5 | 28.27 | 29.9 |
|  | 30DR-100% | 112.2 | 84.8 | 125.4 | 107.2 | 70.9 | 63.7 | 83.9 |
|  | 30DR-0% | 33.73 | 29.07 | 57.53 | 24 | 31.9 | 33.6 | 29.83 |
| Zn (mg/kg) | D0-100% | 10.47 | 10 | 11.1 | 8.75 | 10.07 | 8.73 | 9.77 |
|  | D210-100% | 9.75 | 8.3 | 11.3 | 8.1 | 9.45 | 8.75 | 10.25 |
|  | D210-0% | 10.17 | 10.57 | 10.2 | 6.53 | 10.13 | 7.8 | 8.33 |
|  | 30DR-100% | 11.15 | 11.7 | 10.2 | 9.4 | 9.9 | 8.85 | 9 |
|  | 30DR-0% | 6.6 | 5.17 | 5.63 | 10.8 | 10.3 | 8.17 | 7.55 |

Values are means (*n* = 3 ± standard error) of three independent measurements from three samples for each genotype, i.e. one per tree, obtained by pooling 8 fully-expanded leaves. Data were analysed using ANOVA and Fisher LSD tests (P < 0.05). Scion/rootstock combinations grown in nutrient reference solution (100%) and without nutrient solution (0%) at the beginning of the experiment (D0); 210 days after the start of nutritional deprivation (D210), and after 30 days of recovery (30DR).
