# Supplementary material for: Influence of Rootstock Genotype and Ploidy Level on Common Clementine (Citrus clementina Hort. ex Tan) Tolerance to Nutrient Deficiency
Source: Front Plant Sci. 2021 Apr 8;12:634237. doi: 10.3389/fpls.2021.634237 (PMC8060649; doi:10.3389/fpls.2021.634237)
Supplement: Supplementary Table 3 — Means of gas exchange parameters and chlorophyll content of the seven scion/rootstock combinations. [file Table_3.docx]

**Supplementary** **Table 3.** Means of gas exchange parameters and chlorophyll content of the seven scion/rootstock combinations.

| Parameters | Day | C/PMC4x | C/PMC2x | C/FL4x | C/CM4x | C/CM2x | C/CC4x | C/CC2x |
| --- | --- | --- | --- | --- | --- | --- | --- | --- |
|  | D0-100% | 7.807 | 9.247 | 9.343 | 10.368 | 10.352 | 9.482 | 10.106 |
| *P*_net_ | D210-100% | 8.287 | 5.965 | 9.837 | 7.530 | 9.970 | 8.630 | 6.480 |
|  | D210-0% | 2.279 | 1.175 | 3.138 | 3.366 | 1.535 | 0.561 | 0.337 |
| (μmol CO_2_.m^-2^.s^-1^) | 30DR-100% | 7.653 | 6.166 | 9.013 | 7.713 | 10.243 | 13.917 | 6.743 |
|  | 30DR-0% | 1.699 | 0.314 | 2.235 | 2.700 | 0.625 | 0.779 | 1.679 |
|  | D0-100% | 0.133 | 0.132 | 0.165 | 0.068 | 0.057 | 0.100 | 0.018 |
| *g*_s_ | D210-100% | 0.143 | 0.070 | 0.160 | 0.103 | 0.118 | 0.010 | 0.083 |
|  | D210-0% | 0.054 | 0.015 | 0.043 | 0.040 | 0.011 | 0.001 | 0.007 |
| (mol CO_2_.m^-2^.s^-1^) | 30DR-100% | 0.078 | 0.108 | 0.100 | 0.090 | 0.127 | 0.007 | 0.100 |
|  | 30DR-0% | 0.020 | 0.010 | 0.026 | 0.025 | 0.010 | 0.000 | 0.022 |
|  | D0-100% | 0.867 | 0.920 | 0.788 | 0.904 | 0.344 | 1.350 | 1.140 |
| E | D210-100% | 1.755 | 1.673 | 2.213 | 1.633 | 1.668 | 1.803 | 1.485 |
|  | D210-0% | 0.828 | 0.276 | 0.489 | 0.784 | 0.242 | 0.202 | 0.273 |
| (mmol H_2_O.m^-2^.s^-1^) | 30DR-100% | 1.208 | 2.498 | 1.737 | 1.416 | 1.860 | 2.423 | 1.610 |
|  | 30DR-0% | 0.365 | 0.197 | 0.313 | 0.575 | 0.312 | 0.288 | 0.251 |
|  | D0-100% | 36.150 | 40.883 | 45.950 | 45.133 | 43.867 | 45.217 | 42.950 |
| Chlorophyll | D210-100% | 76.933 | 73.333 | 69.344 | 74.556 | 76.633 | 70.278 | 73.789 |
|  | D210-0% | 10.078 | 9.533 | 15.117 | 46.821 | 12.951 | 9.979 | 9.519 |
| (DUALEX units) | 30DR-100% | 62.456 | 73.822 | 58.800 | 74.033 | 74.000 | 67.733 | 69.578 |
|  | 30DR-0% | 11.492 | 10.557 | 9.643 | 18.804 | 8.436 | 7.654 | 9.254 |

Values are means (*n* = 9 ± standard error) of nine independent measurements from three leaves for each genotype, i.e. one per tree. Data were analysed using ANOVA and Fisher LSD tests (P < 0.05). Scion/rootstock combinations grown in nutrient reference solution (100%) and without nutrient solution (0%) at the beginning of the experiment (D0); 210 days after the start of nutritional deprivation (D210), and after 30 days of recovery (30DR).
