# Supplementary material for: Influence of Rootstock Genotype and Ploidy Level on Common Clementine (Citrus clementina Hort. ex Tan) Tolerance to Nutrient Deficiency
Source: Front Plant Sci. 2021 Apr 8;12:634237. doi: 10.3389/fpls.2021.634237 (PMC8060649; doi:10.3389/fpls.2021.634237)
Supplement: Supplementary Table 4 — Means of chlorophyll a fluorescence parameters of the seven scion/rootstock combinations. [file Table_4.docx]

**Supplementary** **Table 4.** Means of chlorophyll *a* fluorescence parameters of the seven scion/rootstock combinations.

| Parameters | Day | C/PMC4x | C/PMC2x | C/FL4x | C/CM4x | C/CM2x | C/CC4x | C/CC2x |
| --- | --- | --- | --- | --- | --- | --- | --- | --- |
| *F*_v_*/F*_m_ | D0-100% | 0.825 | 0.821 | 0.820 | 0.825 | 0.826 | 0.771 | 0.785 |
|  | D210-100% | 0.803 | 0.842 | 0.818 | 0.850 | 0.847 | 0.840 | 0.812 |
|  | D210-0% | 0.603 | 0.600 | 0.518 | 0.697 | 0.228 | 0.413 | 0.366 |
|  | 30DR-100% | 0.837 | 0.765 | 0.775 | 0.816 | 0.834 | 0.821 | 0.790 |
|  | 30DR-0% | 0.411 | 0.509 | 0.450 | 0.581 | 0.202 | 0.453 | 0.452 |
|  | D0-100% | 31.050 | 28.667 | 30.983 | 31.050 | 29.183 | 25.367 | 26.817 |
| ETR | D210-100% | 35.767 | 35.733 | 34.133 | 26.233 | 32.800 | 34.800 | 33.833 |
|  | D210-0% | 20.494 | 11.006 | 16.043 | 25.499 | 18.598 | 20.428 | 18.169 |
| (µmol.e^-1^.m^-2^.s^-1^) | 30DR-100% | 31.083 | 25.333 | 30.133 | 26.900 | 21.917 | 31.500 | 27.720 |
|  | 30DR-0% | 13.801 | 6.663 | 17.628 | 28.353 | 7.123 | 23.058 | 21.954 |
| ETR/*P*_net_ | D0-100% | 3.977 | 3.100 | 3.316 | 2.995 | 2.819 | 2.675 | 2.654 |
|  | D210-100% | 4.316 | 5.991 | 3.470 | 3.484 | 3.290 | 4.032 | 5.221 |
|  | D210-0% | 9.163 | 9.363 | 5.111 | 7.581 | 12.117 | 36.397 | 61.636 |
|  | 30DR-100% | 4.062 | 4.109 | 3.343 | 3.487 | 2.140 | 2.263 | 4.111 |
|  | 30DR-0% | 8.144 | 21.188 | 7.943 | 10.501 | 11.387 | 29.575 | 13.076 |
| Y(II) | D0-100% | 0.624 | 0.570 | 0.616 | 0.617 | 0.580 | 0.470 | 0.558 |
|  | D210-100% | 0.711 | 0.710 | 0.310 | 0.521 | 0.652 | 0.691 | 0.667 |
|  | D210-0% | 0.408 | 0.160 | 0.369 | 0.496 | 0.370 | 0.489 | 0.449 |
|  | 30DR-100% | 0.618 | 0.503 | 0.338 | 0.535 | 0.436 | 0.626 | 0.637 |
|  | 30DR-0% | 0.267 | 0.204 | 0.176 | 0.314 | 0.426 | 0.157 | 0.425 |
| Y(NPQ) | D0-100% | 0.186 | 0.048 | 0.055 | 0.046 | 0.052 | 0.048 | 0.047 |
|  | D210-100% | 0.047 | 0.044 | 0.046 | 0.652 | 0.039 | 0.079 | 0.248 |
|  | D210-0% | 0.080 | 0.075 | 0.070 | 0.901 | 0.062 | 0.085 | 0.116 |
|  | 30DR-100% | 0.046 | 0.045 | 0.044 | 0.436 | 0.040 | 0.085 | 0.089 |
|  | 30DR-0% | 0.059 | 0.037 | 0.075 | 1.107 | 0.414 | 0.061 | 0.253 |

Values are means (*n* = 9 ± standard error) of nine independent measurements from three leaves for each genotype, i.e. one per tree. Data were analysed using ANOVA and Fisher LSD tests (P < 0.05). Scion/rootstock combinations grown in nutrient reference solution (100%) and without nutrient solution (0%) at the beginning of the experiment (D0); 210 days after the start of nutritional deprivation (D210), and after 30 days of recovery (30DR).
