# Supplementary material for: Influence of Rootstock Genotype and Ploidy Level on Common Clementine (Citrus clementina Hort. ex Tan) Tolerance to Nutrient Deficiency
Source: Front Plant Sci. 2021 Apr 8;12:634237. doi: 10.3389/fpls.2021.634237 (PMC8060649; doi:10.3389/fpls.2021.634237)
Supplement: Supplementary Table 5 — Means of antioxidant parameters of the seven scion/rootstock combinations. [file Table_5.docx]

**Supplementary** **Table 5.** Means of antioxidant parameters of the seven scion/rootstock combinations.

| Parameters | Day | C/PMC4x | C/PMC2x | C/FL4x | C/CM4x | C/CM2x | C/CC4x | C/CC2x |
| --- | --- | --- | --- | --- | --- | --- | --- | --- |
|  | D0-100% | 1.461 | 1.437 | 1.530 | 0.853 | 1.398 | 1.674 | 1.561 |
| Asa | D210-100% | 1.069 | 2.259 | 1.057 | 1.926 | 1.308 | 1.349 | 1.900 |
|  | D210-0% | 4.118 | 4.259 | 4.203 | 4.020 | 3.707 | 4.169 | 3.573 |
| (μmol.g^-1^ FW) | 30DR-100% | 3.506 | 3.909 | 3.601 | 3.150 | 3.409 | 2.414 | 3.959 |
|  | 30DR-0% | 1.777 | 2.244 | 1.811 | 2.202 | 2.179 | 1.779 | 1.647 |
|  | D0-100% | 2.068 | 2.810 | 2.437 | 1.356 | 2.390 | 2.189 | 2.188 |
| Asa/DHA | D210-100% | 1.308 | 10.699 | 0.933 | 2.399 | 1.230 | 1.411 | 2.340 |
|  | D210-0% | 4.570 | 50.882 | 6.007 | 7.577 | 3.579 | 3.579 | 2.541 |
|  | 30DR-100% | 4.035 | 5.440 | 2.693 | 7.388 | 1.391 | 1.326 | 4.240 |
|  | 30DR-0% | 6.097 | 7.703 | 9.060 | 4.832 | 2.548 | 5.120 | 2.154 |
|  | D0-100% | 12.986 | 12.026 | 15.217 | 14.677 | 13.685 | 15.421 | 13.487 |
| Proline | D210-100% | 62.414 | 65.309 | 58.883 | 54.287 | 58.766 | 20.611 | 17.379 |
|  | D210-0% | 28.710 | 33.046 | 23.259 | 20.738 | 14.221 | 7.482 | 4.449 |
| (μmol.g^-1^ FW) | 30DR-100% | 50.289 | 59.545 | 50.925 | 57.364 | 62.466 | 28.245 | 17.885 |
|  | 30DR-0% | 16.143 | 28.522 | 28.671 | 19.791 | 22.425 | 15.139 | 5.705 |
|  | D0-100% | 3.734 | 6.238 | 3.773 | 2.766 | 2.996 | 5.113 | 3.773 |
| SOD | D210-100% | 7.870 | 7.303 | 2.981 | 3.931 | 4.321 | 3.544 | 2.061 |
|  | D210-0% | 4.651 | 5.478 | 2.188 | 2.335 | 3.055 | 2.212 | 1.512 |
| (U.mg^-1^ protein) | 30DR-100% | 4.704 | 4.538 | 4.348 | 2.763 | 3.651 | 3.406 | 3.166 |
|  | 30DR-0% | 1.646 | 2.160 | 1.339 | 1.650 | 1.723 | 1.560 | 0.978 |
|  | D0-100% | 1.309 | 1.052 | 1.318 | 1.414 | 1.010 | 0.382 | 0.941 |
| CAT | D210-100% | 1.751 | 1.173 | 0.374 | 0.703 | 0.922 | 0.510 | 0.830 |
|  | D210-0% | 0.839 | 0.330 | 1.323 | 0.914 | 0.754 | 0.454 | 0.646 |
| (μmol.min^-1^ protein) | 30DR-100% | 0.904 | 0.600 | 0.332 | 0.742 | 0.313 | 0.297 | 0.367 |
|  | 30DR-0% | 0.299 | 0.694 | 0.788 | 0.705 | 0.750 | 1.061 | 0.673 |
|  | D0-100% | 1.742 | 1.564 | 1.310 | 1.487 | 1.633 | 1.187 | 1.161 |
| APX | D210-100% | 0.886 | 1.046 | 0.957 | 1.046 | 1.059 | 0.866 | 0.622 |
|  | D210-0% | 1.758 | 2.022 | 1.497 | 1.169 | 1.409 | 1.497 | 1.181 |
| (μmol.min^-1^ protein) | 30DR-100% | 0.237 | 0.286 | 0.253 | 0.286 | 0.279 | 0.255 | 0.274 |
|  | 30DR-0% | 0.578 | 0.585 | 0.702 | 0.747 | 0.742 | 0.725 | 0.595 |
|  | D0-100% | 43.744 | 46.157 | 22.757 | 22.127 | 19.180 | 19.188 | 21.455 |
| DHAR | D210-100% | 21.209 | 17.643 | 26.993 | 28.126 | 23.667 | 38.949 | 27.693 |
|  | D210-0% | 12.323 | 12.227 | 12.201 | 13.191 | 14.437 | 12.542 | 11.631 |
| (μmol.min^-1^ protein) | 30DR-100% | 33.278 | 32.257 | 28.707 | 39.693 | 28.236 | 28.703 | 28.697 |
|  | 30DR-0% | 12.346 | 13.387 | 13.004 | 16.274 | 16.998 | 14.208 | 13.660 |

All data are presented as mean of three independent measurements for the three biological replicates (*n* = 3, ± standard error). Data were analysed using ANOVA and Fisher LSD tests (P < 0.05). Scion/rootstock combinations grown in nutrient reference solution (100%) and without nutrient solution (0%) at the beginning of the experiment (D0); 210 days after the start of nutritional deprivation (D210), and after 30 days of recovery (30DR).
