# Supplementary material for: Influence of Rootstock Genotype and Ploidy Level on Common Clementine (Citrus clementina Hort. ex Tan) Tolerance to Nutrient Deficiency
Source: Front Plant Sci. 2021 Apr 8;12:634237. doi: 10.3389/fpls.2021.634237 (PMC8060649; doi:10.3389/fpls.2021.634237)
Supplement: Supplementary Table 6 — Means of oxidative markers of the seven scion/rootstock combinations. [file Table_6.docx]

**Supplementary** **Table 6.** Means of oxidative markers of the seven scion/rootstock combinations.

| Parameters | Day | C/PMC4x | C/PMC2x | C/FL4x | C/CM4x | C/CM2x | C/CC4x | C/CC2x |
| --- | --- | --- | --- | --- | --- | --- | --- | --- |
|  | D0-100% | 0.709 | 0.571 | 0.675 | 0.631 | 0.592 | 0.792 | 0.720 |
| DHA | D210-100% | 0.831 | 0.940 | 1.145 | 0.800 | 1.068 | 0.974 | 0.838 |
|  | D210-0% | 1.037 | 0.364 | 0.645 | 0.532 | 1.045 | 1.044 | 1.422 |
| (μmol.g^-1^ FW) | 30DR-100% | 0.868 | 0.807 | 0.782 | 0.427 | 2.529 | 1.875 | 0.958 |
|  | 30DR-0% | 0.300 | 0.272 | 0.201 | 0.490 | 0.847 | 0.362 | 0.762 |
|  | D0-100% | 7.582 | 3.093 | 39.943 | 20.478 | 23.077 | 21.622 | 24.494 |
| MDA leaves | D210-100% | 10.361 | 7.839 | 5.930 | 15.529 | 13.011 | 7.104 | 19.472 |
|  | D210-0% | 22.690 | 16.305 | 9.192 | 19.287 | 34.414 | 25.078 | 58.300 |
| (nmol.g^-1^ FW) | 30DR-100% | 20.572 | 11.240 | 16.864 | 11.798 | 21.359 | 14.902 | 10.967 |
|  | 30DR-0% | 18.206 | 19.153 | 19.292 | 3.410 | 46.563 | 33.232 | 10.112 |
|  | D0-100% | 60.096 | 64.293 | 62.605 | 79.600 | 73.948 | 66.795 | 79.524 |
| H_2_O_2_ leaves | D210-100% | 69.749 | 73.035 | 66.913 | 84.845 | 84.554 | 75.878 | 106.523 |
|  | D210-0% | 70.307 | 65.732 | 72.266 | 85.439 | 65.699 | 59.867 | 71.477 |
| (nmol.g^-1^ FW) | 30DR-100% | 39.238 | 42.589 | 40.901 | 64.415 | 50.993 | 44.502 | 37.899 |
|  | 30DR-0% | 22.091 | 29.131 | 40.533 | 69.568 | 65.169 | 46.950 | 23.914 |
|  | D0-100% | 2.818 | 3.256 | 5.724 | 10.328 | 5.946 | 5.185 | 5.272 |
| MDA roots | D210-100% | 5.744 | 2.833 | 3.219 | 9.403 | 5.975 | 5.124 | 5.316 |
|  | D210-0% | 3.797 | 3.720 | 2.478 | 5.830 | 6.506 | 3.243 | 3.269 |
| (nmol.g^-1^ FW) | 30DR-100% | 5.715 | 2.798 | 3.283 | 11.121 | 5.922 | 5.309 | 5.185 |
|  | 30DR-0% | 2.457 | 4.062 | 2.574 | 8.319 | 5.058 | 3.127 | 8.898 |
|  | D0-100% | 22.271 | 22.201 | 31.459 | 23.793 | 23.425 | 21.224 | 11.536 |
| H_2_O_2_  roots | D210-100% | 19.712 | 21.850 | 30.481 | 22.475 | 23.690 | 20.193 | 10.121 |
|  | D210-0% | 14.055 | 15.360 | 12.192 | 19.464 | 29.470 | 22.899 | 11.436 |
| (nmol.g^-1^ FW) | 30DR-100% | 23.829 | 23.277 | 33.414 | 25.168 | 22.908 | 22.063 | 12.243 |
|  | 30DR-0% | 15.203 | 18.645 | 9.857 | 30.881 | 27.490 | 16.591 | 19.919 |

All data are presented as mean of three independent measurements for the three biological replicates (*n* = 3, ± standard error). Data were analysed using ANOVA and Fisher LSD tests (P < 0.05). Scion/rootstock combinations grown in nutrient reference solution (100%) and without nutrient solution (0%) at the beginning of the experiment (D0); 210 days after the start of nutritional deprivation (D210), and after 30 days of recovery (30DR).
